# Efficacy and Safety of Anti-HER2 Targeted Therapy for Metastatic HR-Positive and HER2-Positive Breast Cancer: A Bayesian Network Meta-Analysis

**DOI:** 10.3390/curroncol30090615

**Published:** 2023-09-15

**Authors:** Xian-Meng Wu, Yong-Kang Qian, Hua-Ling Chen, Chen-Hua Hu, Bing-Wei Chen

**Affiliations:** Department of Epidemiology and Health Statistics, School of Public Health, Southeast University, Nanjing 210009, China; wxm9310@126.com (X.-M.W.); 220223632@seu.edu.cn (Y.-K.Q.); 220223663@seu.edu.cn (H.-L.C.); 220223719@seu.edu.cn (C.-H.H.)

**Keywords:** HR-positive, HER2-positive, metastatic breast cancer, targeted therapy, Bayesian network meta-analysis

## Abstract

Despite the development of HER2-targeted drugs, achieving favorable outcomes for patients with HR+/HER2+MBC remains challenging. This study utilized Bayesian Network Meta-analysis to compare the efficacy and safety of anti-HER2 combination regimens. The primary analysis focused on progression-free survival (PFS), while secondary analyses included objective response rate, overall survival (OS) and the incidence rate of grade 3/4 adverse events (AEs). A comprehensive search across seven databases identified 25 randomized controlled trials for inclusion in this meta-analysis. For patients eligible for endocrinotherapy, our findings revealed that dual-target combined endocrine therapy, such as Her2-mAb+Her2-mAb+Endo (HR = 0.38; 95%CrI: 0.16–0.88) and Her2-mAb+Her2-tki+Endo (HR = 0.45; 95%CrI: 0.23–0.89), significantly improved PFS compared to endocrine therapy alone. According to the surface under the cumulative ranking curves (SUCRAs), Her2-mAb+Her2-mAb+Endo and Her2-mAb+Her2-tki+Endo ranked highest in terms of PFS and OS, respectively. For patients unsuitable for endocrine therapy, anti-HER2 dual-target combined chemotherapy, such as Her2-mAb+Her2-mAb+Chem (HR = 0.76; 95%CrI: 0.6–0.96) and Her2-mAb+Her2-tki+Chem (HR = 0.48; 95%CrI: 0.29–0.81), demonstrated significant improvements in PFS compared to Her2-mAb+Chem. The results were the same when compared with Her2-tki+Chem. According to the SUCRAs, Her2-mAb+Her2-tki+Chem and Her2-mAb+Her2-mAb+Chem ranked highest for PFS and OS, respectively. Subgroup analyses consistently supported these overall findings, indicating that dual-target therapy was the optimal approach irrespective of treatment line.

## 1. Introduction

According to GLOBOCAN 2020 [1], breast cancer has, for the first time, surpassed lung cancer as the most common cancer in women, posing a significant threat to their physical and mental health. While the development of new drugs targeting Human Epidermal growth factor Receptor 2 (HER2) has gradually improved patient outcomes, individualized differences still prevent us from achieving desired prognoses. Approximately 15–20% of breast cancers overexpress HER2, and nearly half of HER2+ breast cancers also express hormone receptors (HRs), such as estrogen receptor positive (ER+) and/or progesterone receptor positive (PR+) [2]. HR+/HER2+ breast cancer represents a unique subtype with distinct clinical presentations, physiological behaviors, and therapeutic sensitivities compared to HER2-overexpressing breast cancer. Over time, HR+/HER2+ patients experience a gradual increase in the risk of recurrence following anti-HER2 treatment. This underscores the pressing need for increased attention, especially for HR+/HER2+ metastatic breast cancer (MBC). Maximizing clinical benefits for these specific subtype patients is an imminent issue that requires resolution.

In the case of progressive, recurrent metastatic breast cancer, treatment options must be selected based on the patient’s condition and prior medications. The 2022 NCCN Breast Cancer v3 guidelines outline systemic therapy principles for ER+/PR+HER2+ recurrent unresectable or stage IV breast cancer. These options include systemic chemotherapy plus anti-HER2-targeted therapy, as well as endocrine therapy with or without anti-HER2-targeted therapy [3]. For patients with HR+/HER2+ metastatic breast cancer (MBC), combining anti-HER2 therapy with chemotherapy has demonstrated survival benefits in advanced systemic therapy. Additionally, endocrine-based therapy has received widespread recommendations. Clinical trials such as TANDEM [4], EGF30008 [5], and eLEcTRA [6], which explored single-target combinations with endocrine therapy, reported progression-free survival (PFS) ranging from approximately 4.8 to 14.1 months and overall survival (OS) ranging from approximately 28.5 to 33.3 months. Studies involving dual-target combinations with endocrine therapy, such as ALTERNATIVE [7] and PERTAIN [8], showed PFS ranging from approximately 5.7 to 18.9 months. Furthermore, beyond anti-HER2 combined with endocrine therapy or chemotherapy, promising therapeutic prospects are emerging for HR+/HER2+MBC through the use of combination drugs, including Cyclin-dependent kinase 4/6 (CDK4/6) inhibitors and mTOR (mammalian target of rapamycin) inhibitors [9].

At present, some clinical trials have been published. However, due to their representation as a small portion of the population with HER2+MBC, these studies had small sample sizes and lack sufficient evidence to establish the efficacy and safety of combination therapy regimens involving HER2-targeted drugs. Moreover, the diverse array of anti-HER2 drugs and combinations complicates direct comparisons of randomized controlled trials (RCTs) in clinical settings, and consensus on superiority or inferiority remains elusive. Additionally, Two published network meta-analyses on anti-HER2+ combination therapy for HER2+ABC are constrained by their exclusive focus on combination chemotherapy, limited HR+ subgroup sample size, and incomplete research indicators, rendering them inadequate for comprehensive analysis [10,11]. Given these limitations, this study aims to employ Bayesian Network Meta-analysis to compare the efficacy and safety of anti-HER2-based combination regimens both directly and indirectly for HR+/HER2+ ABC. We have chosen the Bayesian model because it offers more precise and flexible parameter estimates, addressing issues such as instability and bias often observed in frequentist methods. Additionally, Bayesian methods enjoy widespread endorsement and adoption [12,13]. The goal of this research is to provide dependable evidence-based support for clinical practice.

## 2. Methods

This study is registered with PROSPERO as identifier CRD42022378369. We conducted this systematic review following the PRISMA-2020 guidelines and the PRISMA extension statement for network meta-analysis (PRISMA-NMAs) [14,15].

### 2.1. Search Strategy and Selection Criteria

We systematically searched seven databases, including PubMed, the Cochrane Library, Web of Science, EMBASE, Sinomed, CNKI, and WANFANG, to identify all the relevant literature published between 1 January 2000 and 23 November 2022. Our search terms included the following terms: (Breast neoplasms), (Human epidermal growth factor receptor 2 positive), (Progesterone receptor positive OR Estrogen Receptors positive OR Hormone receptor positive OR Triple positive breast cancer), and (Randomized controlled trial OR controlled study). The combination of these terms was used to search relevant articles. To ensure comprehensiveness, a two-person search approach was employed, and any discrepancies were discussed and resolved. The full search strategy is provided in Supplementary Methods Table S1.

The following eligibility criteria were applied to identify relevant studies: (a) Patients diagnosed with advanced/metastatic breast cancer confirmed by histopathology, with molecular confirmation of ER+ and/or PR+ and HER2+. (b) Studies with a controlled trial design, regardless of the presence of randomization, allocation concealment, or blinding. (c) Studies reporting at least one of the following outcomes: objective response rate (ORR), PFS, OS, and the incidence rate of grade 3/4 adverse events (grade 3/4 AEs). Primary endpoint: PFS measures the duration from randomization or treatment initiation until disease progression or death from any cause. Secondary endpoints: ORR: calculates the percentage of patients in the analysis set who achieved a complete or partial response. OS measures the time from randomization or treatment start to death from any cause. Incidence of adverse events graded as 3 or 4: This endpoint assesses the occurrence and severity of adverse events associated with the treatment. The following criteria led to study exclusion: (a) Ongoing studies without sufficient available data. (b) Meta-analyses, letters, case reports, and reviews. (c) Duplicate publications in different journals. Preference was given to full papers, but in cases where only abstracts with adequate results were available, those studies were included in the analyses. If multiple publications reported results for the same patient cohort, the most recent publication was utilized for analysis. Two investigators used Endnote X9 software for the literature management and employed the software’s de-duplication function to remove duplicate articles. Subsequently, both investigators independently screened the literature at the title, abstract, and full-text levels to determine study inclusion based on the criteria. Inclusion decisions were cross-checked by the two investigators, and in case of any disagreements, a third investigator independently assessed whether to include the study.

### 2.2. Data Extraction and Quality Assessment

Data extraction was conducted independently by two researchers (Qian and Chen). Microsoft Excel was used to record the data from the included studies. The following information was extracted: title, first author, journal name, publication date, the literature type, sample size, age, treatment lines, ER status, PR status, menopausal status, presence of visceral metastasis, drug name, route of administration, dosage, treatment cycle, outcomes, and follow-up period. For studies involving dichotomous variables, we extracted the number of events, the number of non-events, and the total number of events. Continuous variables were extracted as mean and standard deviation. Survival data were expressed as Hazard Ratio (HR) along with their corresponding 95% confidence interval (CI).

Following CONSORT guidelines [16], the quality assessment of randomized controlled studies was conducted using the Revised Cochrane risk-of-bias tool. This tool evaluated several domains of potential bias, including Bias in randomization, Bias in deviation from established intervention, Bias in missing outcome data, Bias in outcome measurement, and Bias in selective reporting outcomes. This rigorous evaluation ensured the methodological quality of the studies and enhanced the reliability of the findings.

### 2.3. Data Synthesis and Statistical Analysis

In this study, statistical analysis was conducted using the following software: StataSE software (version 17.0), RevMan (version 5.0), and R (version 4.2.2) with the ‘getmc’ and ‘rjags’ packages. Studies with treatment groups that could not be linked to other interventions were excluded. Comparison-corrected funnel plots were used to detect publication bias. The random effects model was chosen for analysis, and the error information criterion (DIC) of the random or fixed models was presented. Heterogeneity of results was assessed using I^2^, with I^2^ > 50% and *p* < 0.05 suggesting significant heterogeneity. Multiple treatment options were compared, and the surface under the cumulative ranking curve (SUCRA) was calculated to determine the merits of the treatment options. A higher SUCRA value indicated a better treatment option. If there were no apparent inconsistencies between global and local analyses, the consistency model was selected. Sensitivity analysis was employed to assess the robustness and dependability of the combined outcomes in the meta-analysis. Fitting analysis was performed under fixed and random models for sensitivity analysis. Minimal alteration in the results pre- and post-analysis indicated low sensitivity and stable outcomes. Conversely, significant differences or contradictions in the findings suggested high sensitivity and unstable results. All statistical tests were two-sided, and *p* < 0.05 was considered to denote statistical significance.

### 2.4. Subgroup Analysis

We conducted subgroup analyses for HER2+/HR+ advanced breast cancer based on the number of treatment lines, dividing patients into two groups: ‘treatment line 1’ and ‘treatment line >1’. Our analysis included endpoints such as PFS and OS. ‘Treatment line 1’ was defined as having no prior anti-tumor treatment in the advanced breast cancer stage, while ‘treatment line >1’ was defined as having completed at least one prior systemic anti-tumor treatment in the advanced stage.

## 3. Results

### 3.1. Characteristics of the Included Study

**Figure 1:**
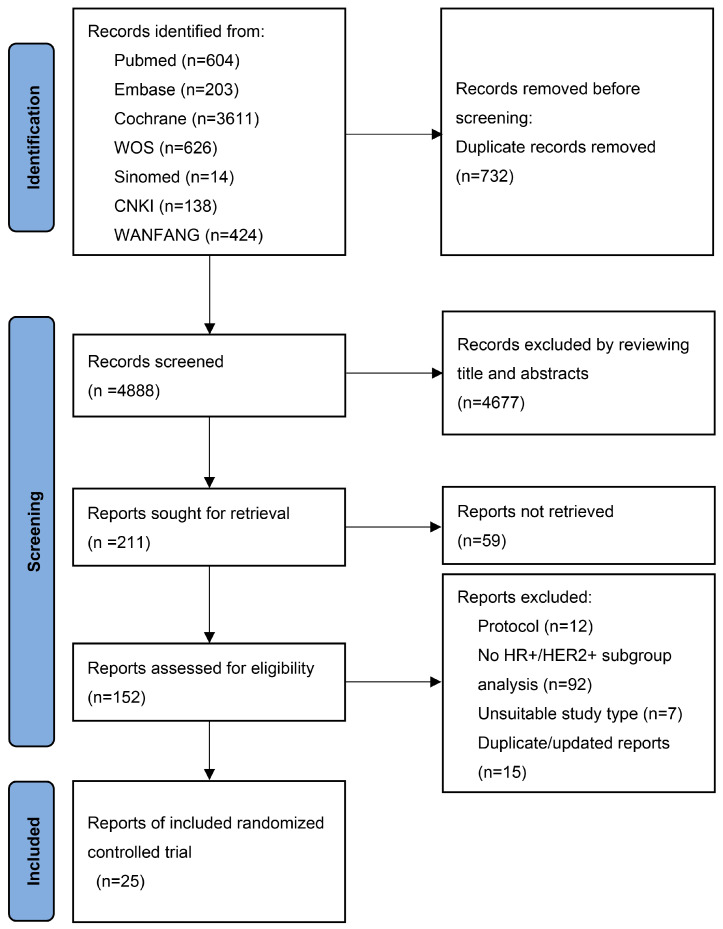
Study selection process based on the PRISMA guideline. PRISMA, Preferred Reporting Items for Systematic Reviews and Meta-analysis.

**Figure 2:**
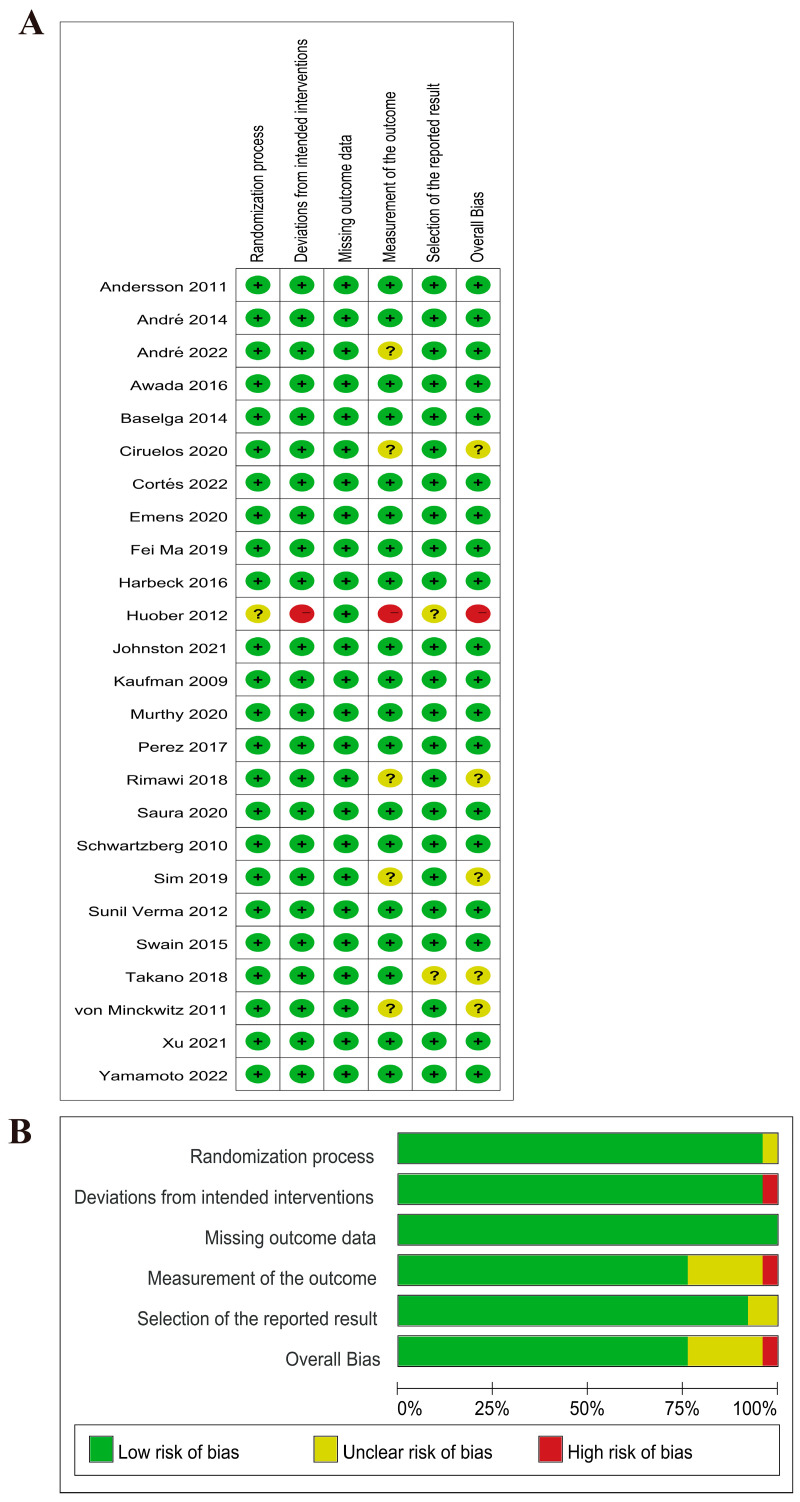
The assessment of bias risk of included studies. (**A**) Bias risk summary. Bias risk was classified as low (+), unclear (?), or high (−). (**B**) Bias risk graph. Reviewing authors’ judgements about the bias risk of each item, and they were shown as percentages across all included studies.

The literature search and screening process was summarized in Figure 1. A total of 5620 articles were initially identified from 7 databases. Ultimately, the network meta-analysis included 25 RCTs, involving a total of 5958 patients. Table 1 provides an overview of the 25 RCTs included in this analysis. All these studies were multi-center, with 17 of them being international multi-center trials. The study design included 3 three-arm trials and 22 two-arm trials. The participants in the studies had a median age of over 50 years. The median follow-up duration was 31 months, ranging from 8.5 to 52.9 months. Among the included studies, 8 (32%) specifically focused on patients with advanced line 1 disease, while 4 studies included patients with line 3 disease or beyond. The remaining studies enrolled patients with at least line 1 disease. Regarding therapeutic interventions, chemotherapy was investigated in 14 studies, while endocrine therapy was examined in 10 studies. Anti-HER2-targeting therapy was evaluated in 25 studies, including monoclonal antibody (mAb), tyrosine kinase inhibitor (TKI), and antibody-drug conjugates (ADC) in 18, 11, and 4 studies, respectively. Other targeted therapies assessed included CDK4/6 inhibitor (2 studies), PD-1 inhibitor (1 study), and mTOR inhibitor (1 study). The quality of the included RCTs was assessed using Cochrane quality assessments, as shown in Figure 2.

### 3.2. Network Meta-Analysis

#### 3.2.1. Network Diagrams

**Figure 3:**
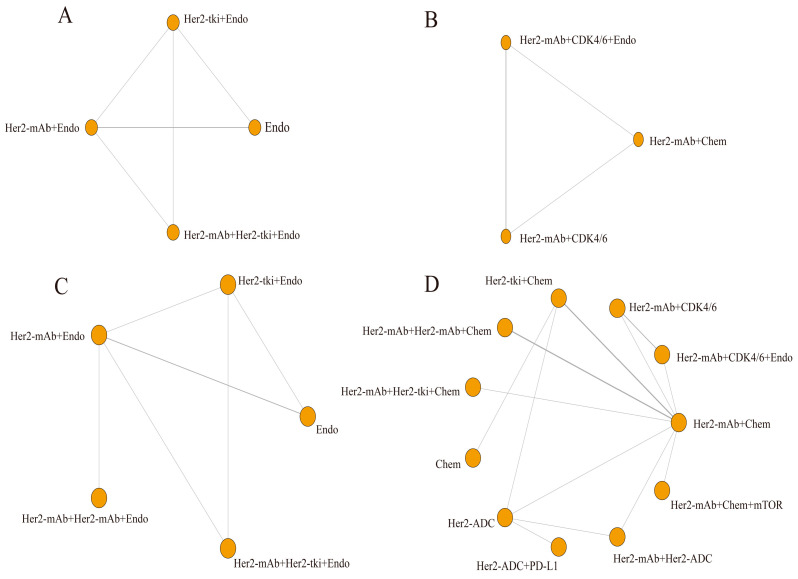
Network plots of each targeted treatment regimen for ORR and PFS (**A**) ORR^#1^; (**B**) ORR^#2^; (**C**) PFS^#1^; (**D**) PFS^#2^. Her2-ADC: trastuzumab deruxtecan, trastuzumab emtansine; Her2-mAb: pertuzumab, trastuzumab; Her2-tki: afatinib, lapatinib, neratinib, pyrotinib, tucatinib; Chem: capecitabine, docetaxel, doxorubicin, paclitaxel, physician’s choice Chemtherapy, taxane, vinorelbine; Endo: anastrozole, fulvestrant, letrozole; CDK/4/6: abemaciclib, palbociclib; mTOR: everolimus; PD-L1: atezolizumab. Each node represents a treatment option, and the lines between them depict direct comparisons in this study. The size of the nodes and the thickness of the lines correspond to the number of direct comparisons made between the treatment options. Larger nodes and thicker lines indicate a higher number of direct comparisons conducted in this study (ORR: objective response rate; PFS: progression free survival).

**Figure 4:**
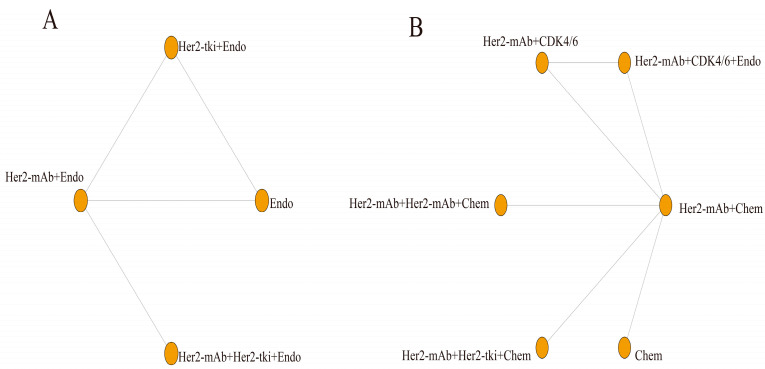
Network plots of each targeted treatment regimen for OS (**A**) OS^#1^; (**B**) OS^#2^. Her2-mAb: pertuzumab, trastuzumab; Her2-tki: afatinib, lapatinib, neratinib, pyrotinib, tucatinib; Chem: capecitabine, docetaxel, doxorubicin, paclitaxel, physician’s choice Chemtherapy, taxane, vinorelbine; Endo: anastrozole, fulvestrant, letrozole; CDK/4/6: abemaciclib, palbociclib. Each node represents a treatment option, and the lines between them depict direct comparisons in this study. The size of the nodes and the thickness of the lines correspond to the number of direct comparisons made between the treatment options. Larger nodes and thicker lines indicate a higher number of direct comparisons conducted in this study (OS: overall survival).

**Figure 5:**
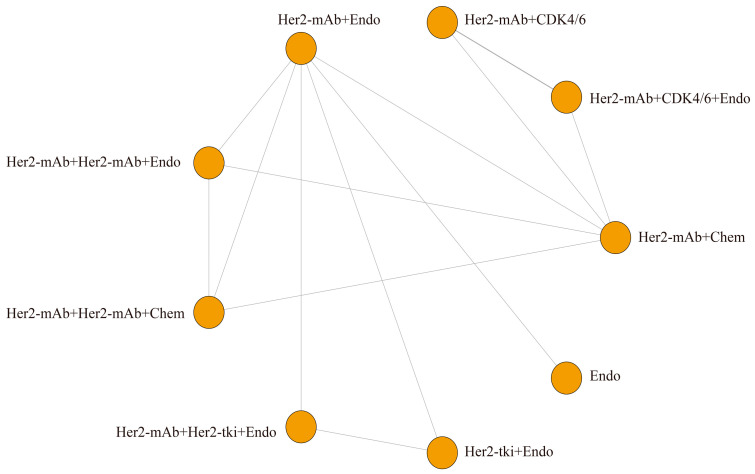
Network plot of each targeted treatment regimen for the grade 3/4 adverse event rate. Her2-mAb:pertuzumab, trastuzumab; Her2-tki: afatinib, lapatinib, neratinib, pyrotinib, tucatinib; Chem: capecitabine, docetaxel, doxorubicin, paclitaxel, physician’s choice Chemtherapy, taxane, vinorelbine; Endo: anastrozole, fulvestrant, letrozole; CDK/4/6: abemaciclib, palbociclib. Each node represents a treatment option, and the lines between them depict direct comparisons in this study. The size of the nodes and the thickness of the lines correspond to the number of direct comparisons made between the treatment options. Larger nodes and thicker lines indicate a higher number of direct comparisons conducted in this study.

This study analyzed four outcome indicators and created network structure diagrams. Due to the absence of direct comparisons between certain treatments, ORR, PFS, and OS were separated into two sections. The results revealed 4 regimens for ORR^#1^ (Figure 3A) and 3 regimens for ORR^#2^ (Figure 3B), 5 regimens for PFS^#1^ (Figure 3C) and 11 regimens for PFS^#2^ (Figure 3D), 4 regimens for OS^#1^ (Figure 4A) and 6 regimens for OS^#2^ (Figure 4B), and 9 regimens for the incidence rate of grade 3/4 AEs (Figure 5). For all indicators, studies on different targeted drugs did not produce an effective closed loop.

#### 3.2.2. Efficacy

Due to the absence of direct comparisons between certain treatments, PFS, OS, and ORR were separated into two sections, namely PFS^#1^ and PFS^#2^, ORR^#1^ and ORR^#2^, OS^#1^ and OS^#2^. ‘1’ represents the combination of anti-Her2 therapy with endocrine treatment, while ‘2’ primarily represents the combination of anti-Her2 therapy with chemotherapy or CDK4/6 inhibitors.

The Bayesian network meta-analysis of PFS was divided into two parts. The results for PFS^#1^ are presented in Table 2 and Figure S1, revealing that double-target combined endocrine therapies, Her2-mAb+Her2-mAb+Endo (HR = 0.38; 95%CrI: 0.16–0.88) and Her2-mAb+Her2-tki+Endo (HR = 0.45; 95%CrI: 0.23–0.89), significantly improved PFS in MBC compared to Endo alone. However, single-target combined endocrine therapies such as Her2-tki+Endo (HR = 0.65; 95%CrI: 0.39–1.06) and Her2-mAb+Endo (HR = 0.69; 95%CrI: 0.45–1.05) did not significantly improve PFS in patients with HR+/HER2+MBC compared to Endo alone. According to the SUCRAs (Figure S2), for PFS^#1^, the treatments can be ranked as follows: Her2-mAb+Her2-mAb+Endo (SUCRA = 87.9%) > Her2-mAb+Her2-tki+Endo (SUCRA = 79.1%) > Her2-tki+Endo (SUCRA = 44.1%) > Her2-mAb+Endo (SUCRA = 36.3%) > Endo (SUCRA = 2.5%).

For PFS^#2^ (Table 3 and Figure S3), Her2-mAb+Her2-mAb+Chem (HR = 0.76; 95%CrI: 0.6–0.96) and Her2-mAb+Her2-tki+Chem (HR = 0.48; 95%CrI: 0.29–0.81) significantly improved PFS compared to Her2-mAb+Chem. Similarly, Her2-mAb+Her2-mAb+Chem (HR = 0.65; 95%CrI: 0.46–0.91) and Her2-mAb+Her2-tki+Chem (HR = 0.41; 95%CrI: 0.23–0.73) demonstrated significant improvements in PFS compared to Her2-tki+Chem. All the above dual-target combined chemotherapy approaches showed notable PFS improvements compared to single-target combined chemotherapy. ADC-containing regimens, such as Her2-ADC (HR = 0.75; 95%CrI: 0.57–1) and Her2-mAb+Her2-ADC (HR = 0.63; 95%CrI: 0.42–0.95), significantly improved PFS in HR+/HER2+ABC compared to HER2-tki+Chem. According to the SUCRAs (Figure S4), the top five rankings for improving patient PFS are: Her2-mAb+Her2-tki+Chem (SUCRA = 95.3%) > Her2-mAb+Her2-ADC (SUCRA = 71.1%) > Her2-mAb+Her2-mAb+Chem (SUCRA = 69.2%) > Her2-mAb+CDK4/6+Endo (SUCRA = 60.0%) > Her2-ADC (SUCRA = 49.0%).

We conducted a Bayesian network meta-analysis to compare OS outcomes of two treatment types: combination endocrine therapy (OS^#1^) and combination CDK4/6 inhibitor or chemotherapy (OS^#2^), as shown in Table 4. For OS^#1^, which included HR+HER2+ABC patients, Her2-tki+Endo, Her2-mAb+Endo, and Her2-mAb+Her2-tki+Endo showed a tendency towards improved OS compared to Endo alone in terms of OS (Figure S5). However, these results did not reach statistical significance. Single-target combined endocrine treatments, such as Her2-tki+Endo and Her2-mAb+Endo, had lower OS benefits compared to the dual-target combined endocrine treatment (Her2-mAb+Her2-tki+Endo), but this difference was not statistically significant. Based on the SUCRAs (Figure S6), the rankings for improving patient OS were as follows: Her2-mAb+Her2-tki+Endo (SUCRA = 92.2%) > Her2-mAb+Endo (SUCRA = 52.2%) > Her2-tki+Endo (SUCRA = 50.5%) > Endo alone (SUCRA = 5.1%).

In our Bayesian network meta-analysis, we assessed the impact of combining CDK4/6 inhibitors or chemotherapy with OS (OS^#2^). Similar to the findings for PFS^#2^, dual-target combination chemotherapy, such as Her2-mAb+Her2-mAb+Chem (HR = 0.71; 95%CrI: 0.43–1.18), and Her2-mAb+Her2-tki+Chem (HR = 0.85; 95%CrI: 0.49–1.47), showed a tendency toward OS benefits compared to Her2-mAb+Chem. However, these differences did not reach statistical significance. Among the other chemotherapy-containing regimens compared to Chem alone, we observed an increasing trend in OS benefit for HR+/HER2+MBC patients, but no significant differences were found (Figure S7). Based on the SUCRAs (Figure S8), the ranking for improving patient OS were as follows: Her2-mAb+Her2-mAb+Chem had the highest probability of ranking first (SUCRA = 72.4%), followed by Her2-mAb+CDK4/6 (SUCRA = 67.0%), Her2-mAb+CDK4/6+Endo (SUCRA = 64.4%), Her2-mAb+Her2-tki+Chem (SUCRA = 50.3%), Her2-mAb+Chem (SUCRA = 26.5%), and Chem alone, which ranked last (SUCRA = 19.3%).

In Table 5, we compared the effects of different treatments on ORR. The results of ORR^#1^ showed that Her2-tki+Endo (OR = 3.26; 95%CrI: 1.13–10.17), Her2-mAb+Endo (OR = 2.81; 95%CrI: 1.08–8.18), and Her2-mAb+Her2-tki+Endo (OR = 7.37; 95%CrI: 1.81–33.72) significantly improved OR compared to Endo (Figure S9). Dual-target combined endocrine therapy (Her2-mAb+Her2-tki+Endo) demonstrated greater benefits in ORR compared to single-target combined endocrine therapy (Her2-mAb+Endo, Her2-tki+Endo), although the difference was not statistically significant. These findings were consistent with PFS^#1^ and OS^#1^ for HR+/HER2+MBC patients, where combined therapy showed better effects compared to endocrine therapy alone, with dual-target combined endocrine therapy being the most effective combination. According to the SUCRAs (Figure S10), Her2-mAb+Her2-tki+Endo had the highest probability of being ranked highest for effectively improving ORR (SUCRA = 95.5%). Her2-tki+Endo ranked second (SUCRA = 56.3%), Her2-mAb+Endo ranked third (SUCRA = 46.7%), and Endo had the lowest probability of effective ORR improvement, ranking last (SUCRA = 1.4%).

For ORR^#2^, Her2-mAb+CDK4/6+Endo and Her2-mAb+CDK4/6 showed a potential increase in ORR compared to Her2-mAb+Chem, but no significant difference was observed (Figure S11). According to the SUCRAs (Figure S12), Her2-mAb+Chem had the highest probability of being ranked highest in terms of effectively improving ORR (SUCRA = 64.6%). Her2-mAb+CDK4/6 ranked second (SUCRA = 62.3%), while Her2-mAb+CDK4/6+Endo had the lowest probability and ranked last (SUCRA = 23.9%) in terms of effectively improving ORR.

#### 3.2.3. Safety

Based on the results in Supplementary Safety Table S2, all eight treatments (Her2-mAb+Chem; Her2-mAb+CDK4/6+Endo; Her2-mAb+CDK4/6; Her2-mAb+Endo; Her2- mAb+Her2-mAb+Endo; Her2-mAb+Her2-mAb+Chem; Her2-mAb+Her2-tki+Endo; Her2-tki+Endo) demonstrated a tendency to increase the occurrence of grade 3/4 AEs compared to Endo alone (Figure S13). However, only the difference in Her2-mAb+Her2-mAb+Chem reached statistical significance. According to the SUCRAs (Figure S14), Endo had the highest probability of being ranked first in terms of reducing the incidence of grade 3/4 AEs (SUCRA = 94.4%). Her2-mAb+Endo ranked second (SUCRA = 72.3%) in reducing the incidence rate of grade 3/4 AEs, followed by Her2-mAb+Her2-mAb+Endo (SUCRA = 69.2%) in third place. Her2-tki+Endo ranked fourth (SUCRA = 52.8%) in reducing the incidence rate of grade 3/4 AEs, while Her2-mAb+Her2-tki+Endo ranked fifth (SUCRA = 49.2%). Among the treatments, Her2-mAb+CDK4/6+Endo was the least effective in reducing the incidence rate of grade 3/4 AEs and ranked last (SUCRA = 15.3%).

#### 3.2.4. Inconsistency

We compared the DIC between the inconsistency model and the consistency model for various indicators. The results, shown in Table 6, indicate that the consistency model generally has lower DIC values compared to the inconsistency model across all metrics. This suggests that the consistency assumption is largely met in the analysis.

#### 3.2.5. Subgroup and Sensitivity Analyses

To investigate the impact of different treatment lines (1 line and > 1 line) on treatment regimens, we conducted a subgroup analysis of PFS and OS. However, due to limitations in the available research, we were unable to perform further analysis of some indicators.

For patients with treatment lines of 1, we focused on analyzing PFS^#1^, PFS^#2^, and OS^#1^. Among these, the SUCRA values indicated that Her2-mAb+Her2-mAb+Endo was the most effective in improving PFS (SUCRA = 94.31%), followed by Her2-mAb+Endo (SUCRA = 54.30%), and then Her2-tki+Endo (SUCRA = 45.13%). Conversely, Endo had the least favorable efficacy and was ranked last (SUCRA = 6.27%). Notably, Endo was significantly inferior to Her2-mAb+Her2-mAb+Endo in improving PFS (HR = 2.83, 95% CI: 1.08–7.32). For OS^#1^ with treatment lines of 1, Her2-mAb+Endo ranked the highest in improving OS (SUCRA = 79.56%), followed by Her2-tki+Endo (SUCRA = 60.41%), while Endo had the least efficacy (SUCRA = 10.04%). However, no significant differences were observed between any of the treatment regimens. For PFS^#2^ with treatment lines of 1, the league table did not show any significant differences among (Table S3) Her2-mAb+Her2-mAb+Chem, Her2-mAb+Her2-ADC, Her2-ADC, and Her2-mAb+Chem in terms of improving PFS. Based on the SUCRA results, Her2-mAb+Her2-mAb+Chem ranked highest in improving PFS (SUCRA = 78.72%), followed by Her2-mAb+Her2-ADC (SUCRA = 73.5%), while Her2-ADC had the second lowest efficacy (SUCRA = 31.19%), and Her2-mAb+Chem ranked the lowest (SUCRA = 16.59%) (Table S1). Unfortunately, due to limitations in the available research data, it was not possible to analyze OS^#2^ with treatment line 1.

In the league table for PFS^#2^ with treatment lines >1, we observed that Her2-mAb+Her2-tki+Chem was superior to Her2-tki+Chem in improving PFS (HR = 0.42, 95% CI: 0.18–0.98) (Table S4). The SUCRA values indicated that Her2-mAb+Her2-tki+Chem had the highest probability and the best effect in improving PFS (SUCRA = 90.6%), followed by Her2-mAb+CDK4/6+Endo (SUCRA = 60.33%), while Her2-ADC ranked third (SUCRA = 57.76%). In comparison, Her2-mAb+Her2-mAb+Chem ranked fourth (SUCRA = 57.28%) (Table S2).

Regarding the indicators ORR^#1^, ORR^#2^, PFS^#1^, PFS^#2^, OS^#1^, OS^#2^, and the incidence rate of grade 3/4 AEs, we performed a sensitivity analysis by comparing the results of the fixed-effect model and the random-effects model. In general, the results for ORR^#2^, PFS^#1^, and the incidence rate of grade 3/4 AEs were not relatively robust, while the results for other indicators were stable (Supplementary Sensitivity Analysis Tables S5–S11).

#### 3.2.6. Small-Study Effects

We assessed the potential impact of small-sample clinical trials on our network meta-analysis and conducted checks for publication bias, particularly to detect exaggerated therapeutic effects in such trials. For this purpose, we utilized a comparison-correction funnel plot, which can be found in Supplementary Small-study Effects Figures S15–S21 depict the funnel plots for ORR, PFS, OS, and the incidence rate of grade 3/4 AEs. The scatter distribution in these funnel plots appears symmetrical, indicating the absence of significant small-sample bias. This observation is further supported by the results of Egger’s test, which show no evidence of publication bias (ORR^#1^: Egger’s P = 0.591; ORR^#2^: Egger’s P = 0.243; PFS^#1^: Egger’s P = 0.584; PFS^#2^: Egger’s P = 0.629; OS^#1^: Egger’s P = 0.754; OS^#2^: Egger’s P = 0.757; incidence rate of grade 3/4 AEs: Egger’s P = 0.763).

## 4. Discussion

Breast cancer has now surpassed lung cancer as the most prevalent cancer, with 2,261,419 new cases reported in 2020. This accounts for 11.7% of the total cancer incidence and 6.9% of female breast cancer-related deaths. HR+/HER2+ breast cancer is a specific subtype of HER2+ breast cancer that exhibits distinct biological and clinical characteristics [2]. In recent years, various treatment approaches have been investigated for HR+/HER2+ metastatic breast cancer, including combinations of anti-HER2 therapies with endocrine therapy and anti-HER2 therapy combined with chemotherapy. However, no studies have specifically compared these different treatment combinations to determine the optimal choice. To address this gap, a network meta-analysis was conducted to compare the efficacy and safety of various anti-HER2 combined therapies for advanced HR+/HER2+ breast cancer. This study aimed to provide a scientific and rational treatment choice by examining the efficacy and safety of different anti-HER2 combined therapies. Through analyzing the PFS outcomes, this study intended to identify the most effective treatment option. Both the FDA (Food and Drug Administration) and EMA (European Medicines Agency) consider significant improvements in PFS as a crucial factor in determining the approval of new drugs. Additionally, OS, ORR, and the occurrence of the incidence rate of grade 3/4 AEs were assessed as secondary endpoints to provide a more comprehensive evaluation of treatment efficacy and safety.

In our comprehensive analysis of 25 multi-center RCTs, we evaluated the efficacy of different treatment regimens for HR+/HER2+MBC patients. Specifically, we focused on the combination of anti-HER2 therapy with endocrine therapy (PFS^#1^/OS^#1^/ORR^#1^). Our findings revealed that the combination therapy consistently improved outcomes compared to mono-endocrine therapy. Notably, the combination of dual-target therapy with endocrine therapy emerged as the most effective treatment combination. Among the various regimens analyzed, Her2-mAb+Her2-mAb+Endo showed the highest SUCRA value in the context of PFS^#1^, suggesting it to be the optimal treatment option. Additionally, the SUCRA values for ORR^#1^ and OS^#1^ indicated that Her2-mAb+Her2-tki+Endo was the preferred treatment, alongside Her2-mAb+Her2-mAb+Endo. Therefore, the combination of anti-HER2 therapy with endocrine therapy, particularly utilizing dual-target therapy, demonstrated the best outcomes in terms of prolonging PFS and OS in HR+/HER2+MBC patients.

In terms of anti-HER2 combined chemotherapy or CDK4/6 inhibitors (PFS^#2^/OS^#2^/ORR^#2^), the top four treatment regimens included Her2-mAb+Her2-mAb+Chem, Her2-mAb+CDK4/6+Endo, and Her2-mAb+Her2-tki+Chem, although their specific order varied depending on the outcome measure (OS^#2^ or PFS^#2^). These results suggest that these three-drug combinations can effectively prolong PFS and OS in HR+/HER2+MBC and are thus preferred treatment options. Considering safety, the SUCRA results indicated that Her2-mAb+CDK4/6+Endo had the lowest ranking. Therefore, for anti-HER2 combined chemotherapy or CDK4/6 inhibitors, Her2-mAb+Her2-mAb+Chem and Her2-mAb+Her2-tki+Chem are the preferred options. Overall, the findings from this study highlight the superiority of anti-HER2 therapy combined with endocrine therapy or chemotherapy, with dual-target treatment combinations showing the best outcomes. This holds true for both the combination of two monoclonal antibodies and the combination of a monoclonal antibody with a tyrosine kinase inhibitor.

In this study, only trastuzumab and pertuzumab were utilized as large molecule double-target therapies. Mechanistically, trastuzumab and pertuzumab target distinct epitopes of the HER-2 tyrosine kinase receptor. Trastuzumab promotes HER-2 dimerization inhibition and disrupts downstream Akt signaling, leading to increased apoptosis [37]. These combined effects synergistically inhibit the survival of HER2+ breast cancer cells [38]. In the international multi-center randomized Phase II PERTAIN study, postmenopausal patients with HER2-positive HR-positive locally advanced or metastatic breast cancer (n = 258) who had not previously received endocrine therapy were divided into two groups. One group received pertuzumab plus trastuzumab plus aromatase inhibitor (AI) as first-line treatment, while the other group received trastuzumab plus AI alone. The results demonstrated that the three-drug combination significantly improved PFS compared to the two-drug combination (18.9 months vs. 15.8 months; HR = 0.65, 95% CI: 0.48–0.89) [8]. In HER2-positive and HR-positive breast cancer, aromatase inhibitors are more susceptible to resistance due to HER2 pathway activation. Therefore, a possible explanation for the increased PFS observed in this study is that pertuzumab effectively blocks the HER2 signaling pathway, thereby reducing resistance and improving treatment outcomes. In another Phase III study called CLEOPATRA, 808 women with HER2-positive advanced breast cancer were enrolled, and the efficacy of pertuzumab combined with trastuzumab and docetaxel was compared to trastuzumab combined with docetaxel as the first-line treatment. The combination of docetaxel, trastuzumab, and pertuzumab demonstrated improved mPFS compared to the placebo (18.5 months vs. 12.4 months; HR = 0.62, 95% CI: 0.51–0.75). Subgroup analysis specifically in HR-positive patients showed that pertuzumab was associated with a 27% lower risk of recurrence of advanced first-line breast cancer and a 30% lower risk of death compared to placebo. The findings from multiple clinical studies, such as PERTAIN, CLEOPATRA [39], and PERUSE [40], consistently confirmed that targeting HER2-positive breast cancer with two different monoclonal antibodies, pertuzumab and trastuzumab, leads to a more comprehensive blockade of HER2 signaling compared to treatment with either antibody alone. The more favorable blockade was caused by the complementary mechanisms of action for pertuzumab and trastuzumab. These studies highlighted the significance of preventing ligand-dependent formation of HER2 dimers to maximize the suppression of HER2 signaling.

Monoclonal antibodies and tyrosine kinase inhibitors targeting ErbB2 (HER2) exhibited distinct mechanisms of action and are non-cross-resistant. Studies have demonstrated that combining lapatinib, a tyrosine kinase inhibitor, with anti-ErbB2 antibodies enhanced apoptosis in breast cancer cells that overexpress ErbB2. This combination also modulated the expression of mediators involved in tumor cell growth and survival. The inhibition of phosphorylated Akt (p-Akt) by lapatinib and anti-ErbB2 antibodies is associated with the disruption of ErbB3 activation. It is likely that lapatinib targets other molecules besides full-length ErbB1 and ErbB2 [41]. Recent findings indicated that lapatinib effectively inhibited signaling through the truncated ErbB2 receptor, p95ErbB2 [42]. Although antibodies alone did not inhibit signaling through p95ErbB2, they reduced truncation by inhibiting the proteolytic cleavage of full-length ErbB2. In the ALTERNATIVE study, which focused on postmenopausal patients with HER2+/HR+ metastatic breast cancer (n = 355), participants were randomly assigned in a 1:1:1 ratio to receive trastuzumab plus lapatinib plus AI, lapatinib plus AI, or trastuzumab plus AI. The PFS in the lapatinib plus AI group and the trastuzumab plus AI group were 8.3 months and 5.6 months, respectively (HR = 0.85, 95% CI: 0.62–1.17; *p* = 5.3159). Compared to the trastuzumab plus AI group, the three-drug combination group (lapatinib plus trastuzumab plus AI) demonstrated a significantly prolonged PFS (11 months vs. 5.7 months; HR = 0.62, 95% CI: 0.45–0.88, *p* = 0.0064) [7]. These results suggest that the efficacy of dual-target combined with AI is superior to single-target combined with AI, with comparable safety except for grade 3 diarrhea. The combination of anti-HER2 monoclonal antibody and tyrosine kinase inhibitor is consistent with the findings of our study, both in terms of the mechanism of action and clinical data.

Based on the guidelines, patients eligible for endocrine therapy, such as those with non-visceral metastasis and non-endocrine refractory patients, can be recommended an anti-HER2 dual-target combination endocrine treatment strategy as the first option. Alternatively, for patients who are not suitable for endocrine therapy, anti-HER2 dual-target combination chemotherapy can be chosen as a treatment approach.

Based on the subgroup analysis results considering different treatment lines, we found that among the HR+/HER2+MBC patients in the recurrent metastatic stage who had not received prior systemic treatment (treatment line 1) and were suitable for endocrine therapy, Her2-mAb+Her2-mAb+Endo demonstrated the most favorable outcomes in PFS. For HR+/HER2+MBC patients in the recurrent metastatic stage who had not received prior systemic treatment and were not suitable for endocrine therapy, Her2-mAb+Her2-mAb+Chem showed the best results in improving OS. Among patients who had completed first-line treatment, Her2-mAb+Her2-tki+Chem exhibited superior efficacy in enhancing PFS compared to Her2-mAb+Her2-mAb+Chem, making it the optimal treatment regimen.

The mechanism of action of TKIs complements that of mAbs and can, to some extent, improve resistance to trastuzumab. One advantage of TKIs is that they are small molecules, unlike trastuzumab and pertuzumab, which makes them more suitable for entering the central nervous system (CNS) and potentially better at controlling MBC [43]. In patients past first-line treatment, there are more metastatic lesions and a higher proportion of brain metastases. Therefore, Her2-mAb+Her2-tki+Chem is more suitable in such cases compared to using two Her2-mAbs and Chem. Tucatinib, an oral and highly selective inhibitor of HER2 tyrosine kinase, was examined in the HER2CLIMB study. It was administered alongside trastuzumab and capecitabine to patients with HER2-positive metastatic breast cancer who had received trastuzumab, pertuzumab, and trastuzumab emtansine. In patients with brain metastases, the tucatinib-combination group achieved a 1-year PFS rate of 24.9%. The median PFS for these patients was 7.6 months, which represents an extension of 2.2 months compared to the placebo group. This improvement in brain metastasis control is advantageous for prolonging overall survival [25]. Previous studies have shown some efficacy in treating brain metastases from HER2-positive breast cancer by combining a HER2 tyrosine kinase inhibitor with capecitabine [35,44].

This study found that regimens containing ADC drugs, such as Her2-ADC and Her2-mAb+Her2-ADC, significantly improved PFS in HR+/HER2+MBC patients when compared to Her2-tki+Chem. HER2-mAb+Her2-ADC ranked second and Her2-ADC ranked fifth in providing PFS based on SUCRA values. Antibody-drug conjugates (ADCs) combine the strong cytotoxicity of small molecule drugs with the selectivity of monoclonal antibodies and exhibited good pharmacokinetic characteristics, making them very promising drugs for the treatment of HER2-positive cancers. T-DM1 was the first HER2-targeted ADC approved by the FDA for the treatment of HER2+BC. In 2019, T-DXd (DS-8201) became the second HER2-targeted ADC approved by the FDA, demonstrating significant antitumor activity in patients with refractory HER2+MBC in the interim analysis of DESTINY-Breast03 [45]. In comparison to T-DM1, DS8201 showed a 72% reduction in the risk of disease progression or death (HR = 0.28; 95% CI: 0.22–0.37; *p* < 0.05). In the subgroup analysis of HR+/HER2+MBC, there was a 68% reduction in the risk of progression or death from TDM-1 of 0.32 (0.22–0.46). However, the DESTINY-Breast03 study was not included in this analysis due to treatment classification. Based on the current data, T-DXd is expected to be a very promising drug for the treatment of HER2-positive breast cancer [33].

Although this study followed strict evaluation criteria for network meta-analysis to minimize the impact of confounding factors, there are still some limitations to consider. Despite including 25 RCTs, most of which were international multi-center studies, HR positive was required for the subgroup of HER2-positive patients included in this analysis. Some of the studies in HER2+MBC did not cover ORR, OS and safety specifically for HR+/HER2+MBC, making comprehensive comparisons challenging. Additionally, due to limitations in sample size and available study data, it was not possible to compare specific drugs within the anti-HER2 category. Subgroup analysis based on factors such as site and number of metastatic lesions or the menopausal status of the study population was also not feasible, resulting in some degree of clinical heterogeneity. Furthermore, 19 studies were open label, raising the risk of bias. Therefore, the results should be interpreted cautiously, given the potential subjectivity. Finally, the results of sensitivity analysis indicated inconsistencies between the random effects model and the fixed effects model, suggesting that the findings may not be robust and further confirmation by a high-level clinical trial evidence is necessary.

## 5. Conclusions

In conclusion, the findings suggest that anti-HER2 dual-target combination therapy with endocrine or chemotherapy provides improved survival outcomes for patients with HR+/HER2+MBC compared to anti-HER2 single-target combination therapy. However, it is important to note that the existing research literature is limited, and further studies are needed to gather more data and investigate the comparative efficacy of specific drugs.

## Figures and Tables

**Table 1:** Basic characteristics of the included studies.

Author/Year	Trial Name	Study Design	Median Follow-Up (m)	Total pts	Treatment-Line	Median Age	Intervention	Control	Main Outcome
André 2022 [17]	monarcHER	O, R, P2	52.9	237	≥3	55.3	Group 1: Abemaciclib+Trastuzumab+Fulvestrant Group 2: Abemaciclib+Trastuzumab	Trastuzumab+Standard of Care Single Agent Chemotherapy	ORR, PFS, OS, Safety
Schwartzberg 2010 [5]	EGF30008	DB, R, P3	22.8	219	1	60	Letrozole+Lapatinib	Letrozole+Placebo	ORR, PFS, OS, Safety
Takano 2018 [18]	WJOG6110B/ELTOP	O, R, P2	44.6	54	≥1	58	Trastuzumab+Capecitabine	Lapatinib+Capecitabine	PFS
Awada 2016 [19]	NEfERT-T	O, R, P2	23	251	≥1	55	Neratinib+Paclitaxel	Trastuzumab+Paclitaxel	PFS
Harbeck 2016 [20]	LUX-Breast 1	O, R, P3	9.3	147	1–2	52.2	Afatinib+Vinorelbine	Trastuzumab+Vinorelbine	PFS
Rimawi 2018 [8]	PERTAIN	O, R, P2	31	258	1	61.6	Group 1: Pertuzumab+Trastuzumab+Docetaxel/Paclitaxel→AI Group 2: Pertuzumab+Trastuzumab+AI Group 3: Trastuzumab+Docetaxel/Paclitaxel→ AI	Trastuzumab+AI	PFS, Safety
Swain 2015 [21]	CLEOPATRA	DB, R, P3	50	388	1	53.5	Pertuzumab+Trastuzumab+Docetaxel	Placebo+Trastuzumab+Docetaxel	PFS, OS, Safety
Andersson 2011 [22]	HERNATA	O, R, P3	34	161	1	56	Docetaxel+Trastuzumab	Vinorelbine+Trastuzumab	PFS
Baselga 2014 [23]	STM01-102	O, R, P3	31	156	1	53	Nonpegylated liposomal doxorubicin+Trastuzumab+Paclitaxel	Trastuzumab+Paclitaxel	PFS, OS
Johnston 2021 [7]	ALTERNATIVE	M, O, R, P3	NA	355	1–5	57	Group 1: Trastuzumab+Lapatinib+AI Group 2: Lapatinib+AI	Trastuzumab+AI	ORR, PFS, OS, Safety
Fei Ma 2019 [24]	NA	M, R, P2	14.9	80	1–3	48	Pyrotinib+Capecitabine	Lapatinib+Capecitabine	PFS
Murthy 2020 [25]	HER2CLIMB	M, DB, R, P2	14	289	≥2	55	Tucatinib+Trastuzumab+Capecitabine	Placebo+Trastuzumab+Capecitabine	PFS, OS
Sim 2019 [26]	KCSG BR11-16	M, O, R, P2	NA	59	≥2	53	Lapatinib+Vinorelbine	Vinorelbine	PFS
von Minckwitz 2011 [27]	GBG 26/BIG 3-05	M, O, R, P3	20.7	85	≥2	NA	Trastuzumab+Capecitabine	Capecitabine	OS
Sunil Verma 2012 [28]	EMILIA	M, O, R, P3	13	991	≥1	53	T-DM1	Lapatinib+Capecitabine	PFS
Emens 2020 [29]	KATE2	M, DB, R, P2	8.5	117	≥1	54	T-DM1+Atezolizumab	T-DM1+Placebo	PFS
Perez 2017 [30]	MARIANNE	M, O, R, P3	35	600	1	53	Group 1: T-DM1+Pertuzumab Group 2: T-DM1	Trastuzumab+Taxane	PFS
Kaufman 2009 [4]	TAnDEM	M, O, R, P3	NA	207	1	55	Trastuzumab+Anastrozole	Anastrozole	PFS, OS
Xu 2021 [31]	PHOEBE	M, O, R, P3	33.2	120	≥1	49.5	Pyrotinib+Capecitabine	Lapatinib+Capecitabine	PFS, OS
Ciruelos 2020 [32]	SOLTI-1303 PATRICIA	M, O, R, P2	42.3	56	3–5	58	Trastuzumab+Palbociclib+Letrozole	Trastuzumab+Palbociclib	ORR, PFS, OS, Safety
Cortés 2022 [33]	DESTINY-Breast03	M, O, R, P3	16.2	265	≥1	54.2	Trastuzumab Deruxtecan	Trastuzumab Emtansine	PFS
Yamamoto 2022 [34]	PRECIOUS	M, O, R, P3	14.2	122	3–6	58.5	Pertuzumab+Trastuzumab+physician’s choice chemotherapy (PTC)	Trastuzumab+PTC	PFS
Saura 2020 [6,35]	NALA	M, O, R, P3	29.9	367	≥3	54.4	Neratinib+Capecitabine	Lapatinib+Capecitabine	PFS, OS
Huober 2012 [6]	eLEcTRA	M, O, R, P3	NA	57	1	62	Letrozole+Trastuzumab	Letrozole	ORR, PFS, OS, Safety
André 2014 [36]	BOLERO-3	M, DB, R, P3	20.2	317	≥1	54.2	Everolimus+Trastuzumab+Vinorelbine	Placebo+Trastuzumab+Vinorelbine	PFS

Abbreviations: O: open-label; R: randomized; P: phase; M: multicenter; DB: double blind; S: single arm; NA: not available; OS: overall survival; PFS: progression free survival; ORR: objective response rate; Safety only includes grade 3/4 adverse events.

**Table 2:** Random effects model Bayesian network meta-analysis of estimated HR values for PFS^#1^ between different treatment regimens.

Endo	0.65 (0.39, 1.06)	0.69 (0.45, 1.05)	**0.38 (0.16, 0.88)**	**0.45 (0.23, 0.89)**
1.55 (0.94, 2.55)	Her2-tki+Endo	1.06 (0.64, 1.76)	0.58 (0.25, 1.41)	0.69 (0.38, 1.28)
1.46 (0.95, 2.24)	0.94 (0.57, 1.55)	Her2-mAb+Endo	0.55 (0.27, 1.13)	0.65 (0.35, 1.21)
**2.66 (1.14, 6.14)**	1.71 (0.71, 4.06)	1.82 (0.88, 3.71)	Her2-mAb+Her2-mAb+Endo	1.19 (0.46, 3.05)
**2.23 (1.13, 4.39)**	1.44 (0.78, 2.63)	1.53 (0.83, 2.82)	0.84 (0.33, 2.16)	Her2-mAb+Her2-tki+Endo

Note: The estimated HR and 95%CrI for PFS^#1^ between treatment regimens are shown in the table above, with the lower left corner representing each column treatment group compared to each row treatment group, and the opposite in the upper right corner. HR < 1 indicates that the column treatment regimen prolongs PFS compared to the row treatment regimen; HR > 1 indicates that the column treatment regimen has a poorer benefit on PFS compared to the row treatment regimen. Significant differences between the two groups are shown in bold. (PFS: progression free survival; HR: hazard ratio; CrI: credible interval).

**Table 3:** Random-effects model Bayesian network meta-analysis of estimated HR values for PFS^#2^ between different treatment regimens.

Her2-mAb+Chem	0.8 (0.49, 1.31)	0.95 (0.59, 1.58)	1.17 (0.91, 1.51)	**0.76** **(0.6, 0.96)**	**0.48** **(0.29, 0.81)**	0.95 (0.35, 2.54)	0.88 (0.66, 1.19)	0.95 (0.47, 1.94)	0.74 (0.51, 1.08)	0.93 (0.64, 1.35)
1.25 (0.76, 2.04)	Her2-mAb+CDK4/6+Endo	1.19 (0.78, 1.84)	1.46 (0.85, 2.57)	0.94 (0.55, 1.64)	0.6 (0.29, 1.23)	1.18 (0.4, 3.52)	1.1 (0.63, 1.97)	1.18 (0.5, 2.88)	0.92 (0.5, 1.73)	1.16 (0.63, 2.15)
1.05 (0.63, 1.69)	0.84 (0.54, 1.28)	Her2-mAb+CDK4/6	1.22 (0.71, 2.12)	0.79 (0.46, 1.36)	0.5 (0.25, 1.02)	0.99 (0.33, 2.98)	0.92 (0.52, 1.62)	0.99 (0.42, 2.39)	0.77 (0.42, 1.41)	0.98 (0.53, 1.78)
0.85 (0.66, 1.09)	0.69 (0.39, 1.18)	0.82 (0.47, 1.41)	Her2-tki+Chem	**0.65** **(0.46, 0.91)**	**0.41** **(0.23, 0.73)**	0.81 (0.31, 2.07)	**0.75** **(0.57, 1)**	0.81 (0.4, 1.65)	**0.63** **(0.42, 0.95)**	0.8 (0.51, 1.24)
**1.32** **(1.04, 1.66)**	1.06 (0.61, 1.81)	1.26 (0.74, 2.19)	**1.54** **(1.1, 2.18)**	Her2-mAb+Her2-mAb+Chem	0.63 (0.36, 1.12)	1.25 (0.45, 3.47)	1.16 (0.8, 1.7)	1.26 (0.59, 2.64)	0.97 (0.63, 1.51)	1.23 (0.79, 1.91)
**2.08** **(1.24, 3.47)**	1.68 (0.81, 3.41)	1.99 (0.98, 4.08)	**2.44** **(1.36, 4.3)**	1.58 (0.89, 2.77)	Her2-mAb+Her2-tki+Chem	1.96 (0.64, 6.03)	1.84 (1.01, 3.31)	1.99 (0.81, 4.75)	1.54 (0.81, 2.89)	**1.95** **(1.03, 3.62)**
1.06 (0.39, 2.84)	0.85 (0.28, 2.52)	1.01 (0.34, 3.02)	1.24 (0.48, 3.22)	0.8 (0.29, 2.21)	0.51 (0.17, 1.55)	Chem	0.93 (0.35, 2.53)	1 (0.31, 3.27)	0.78 (0.28, 2.19)	0.99 (0.34, 2.84)
1.13 (0.84, 1.52)	0.91 (0.51, 1.59)	1.08 (0.62, 1.91)	**1.33** **(1, 1.76)**	0.86 (0.59, 1.25)	**0.54** **(0.3, 0.99)**	1.07 (0.4, 2.85)	Her2-ADC	1.08 (0.56, 2.07)	0.84 (0.57, 1.22)	1.06 (0.65, 1.69)
1.05 (0.52, 2.13)	0.84 (0.35, 1.98)	1.01 (0.42, 2.38)	1.23 (0.61, 2.5)	0.8 (0.38, 1.68)	0.5 (0.21, 1.23)	1 (0.31, 3.22)	0.93 (0.48, 1.77)	Her2-ADC+PD-L1	0.78 (0.37, 1.64)	0.98 (0.44, 2.17)
1.36 (0.93, 1.97)	1.09 (0.58, 2)	1.3 (0.71, 2.4)	**1.59** **(1.06, 2.39)**	1.03 (0.66, 1.6)	0.65 (0.35, 1.24)	1.28 (0.46, 3.6)	1.19 (0.82, 1.75)	1.29 (0.61, 2.74)	Her2-mAb+Her2-ADC	1.26 (0.74, 2.13)
1.07 (0.74, 1.56)	0.86 (0.47, 1.58)	1.02 (0.56, 1.9)	1.25 (0.81, 1.98)	0.81 (0.52, 1.27)	**0.51** **(0.28, 0.97)**	1.01 (0.35, 2.96)	0.95 (0.59, 1.54)	1.02 (0.46, 2.29)	0.79 (0.47, 1.35)	Her2-mAb+Chem+mTOR

Note: The estimated HR and 95%CrI for PFS^#2^ between treatment regimens are shown in the table above, with the lower left corner representing each column treatment group compared to each row treatment group, and the opposite in the upper right corner. HR < 1 indicates that the column treatment regimen prolongs PFS compared to the row treatment regimen; HR > 1 indicates that the column treatment regimen has a poorer benefit on PFS compared to the row treatment regimen. Significant differences between the two groups are shown in bold. (PFS: progression free survival; HR: hazard ratio; CrI: credible interval).

**Table 4:** Random effects model Bayesian network meta-analysis OS estimation of HR (A: OS^#1^; B: OS^#2^).

(A) OS^#1^						
Endo	0.69 (0.4, 1.17)		0.67 (0.4, 1.16)		0.4 (0.16, 1.04)	
1.45 (0.86, 2.5)	Her2-tki+Endo		0.97 (0.56, 1.75)		0.59 (0.23, 1.55)	
1.49 (0.86, 2.51)	1.03 (0.57, 1.78)		Her2-mAb+Endo		0.6 (0.28, 1.3)	
2.47 (0.96, 6.21)	1.7 (0.64, 4.34)		1.66 (0.77, 3.56)		Her2-mAb+Her2-tki+Endo	
(B) OS^#2^						
Her2-mAb+Chem	0.75 (0.41, 1.38)	0.73 (0.4, 1.34)	0.71 (0.43, 1.18)	0.85 (0.49, 1.47)		1.11 (0.65, 1.92)
1.33 (0.72, 2.46)	Her2-mAb+CDK4/6+Endo	0.98 (0.54, 1.78)	0.95 (0.43, 2.09)	1.14 (0.5, 2.58)		1.48 (0.66, 3.38)
1.36 (0.74, 2.47)	1.02 (0.56, 1.86)	Her2-mAb+CDK4/6	0.97 (0.44, 2.11)	1.16 (0.52, 2.6)		1.51 (0.67, 3.39)
1.4 (0.85, 2.32)	1.05 (0.48, 2.32)	1.03 (0.47, 2.25)	Her2-mAb+Her2-mAb+Chem	1.2 (0.57, 2.51)		1.56 (0.75, 3.28)
1.17 (0.68, 2.02)	0.88 (0.39, 2)	0.86 (0.38, 1.93)	0.83 (0.4, 1.76)	Her2-mAb+Her2-tki+Chem		1.3 (0.6, 2.81)
0.9 (0.52, 1.55)	0.68 (0.3, 1.52)	0.66 (0.29, 1.48)	0.64 (0.31, 1.33)	0.77 (0.36, 1.65)		Chem

Note: The estimated HR and 95%CrI for OS between treatment regimens are shown in the table above, with the lower left corner representing each column treatment group compared to each row treatment group and the opposite in the upper right corner. HR < 1 indicates that the column treatment regimen prolongs patient OS compared to the row treatment regimen; HR > 1 indicates that the column treatment regimen has a poorer benefit on OS compared to the row treatment regimen. Significant differences between the two groups are shown in bold. (OS: Overall survival; HR: hazard ratio; CrI: credible interval).

**Table 5:** Random effects model Bayesian network meta-analysis of ORR estimation between different treatment regimens OR (A: ORR^#1^; B: ORR^#2^).

(A) ORR^#1^				
Endo	3.26 (1.13, 10.17)	**2.81 (1.08, 8.18)**		**7.37 (1.81, 33.72)**
**0.31 (0.1, 0.89)**	Her2-tki+Endo	0.86 (0.3, 2.65)		2.24 (0.64, 8.24)
**0.36 (0.12, 0.92)**	1.17 (0.38, 3.34)	Her2-mAb+Endo		2.63 (0.71, 9.42)
**0.14 (0.03, 0.55)**	0.45 (0.12, 1.55)	0.38 (0.11, 1.4)		Her2-mAb+Her2-tki+Endo
(B) ORR^#2^				
Her2-mAb+Chem	0.7 (0.25, 1.88)		0.97 (0.36, 2.59)	
1.44 (0.53, 3.98)	Her2-mAb+CDK4/6+Endo		1.38 (0.58, 3.41)	
1.03 (0.39, 2.82)	0.72 (0.29, 1.72)		Her2-mAb+CDK4/6	

Note: The OR and 95%CrI for ORR between treatment regimens are shown in the table above, with the lower left corner representing each column treatment group compared to each row treatment group and the opposite in the upper right corner. OR > 1 indicates that the column treatment regimen improved the ORR of patients compared to the row treatment regimen; OR < 1 indicates that the column treatment regimen had a poorer benefit on ORR than the row treatment regimen. Significant differences between the two groups are shown in bold. (ORR: objective response rate; OR: odds ratio; CrI: credible interval).

**Table 6:** The DIC value of the inconsistency model and the consistency model for various indicators.

Indicators	DIC of Inconsistency Model	DIC of Consistency Model
PFS^#1^	10.865	10.213
PFS^#2^	26.618	24.799
OS^#1^	7.960	7.132
OS^#2^	9.988	9.954
ORR^#1^	18.318	16.166
ORR^#2^	10.141	8.564
Grade 3/4 AEs	28.848	27.587

## Data Availability

The data presented in this study are available on request from the corresponding author.

## Supplementary Materials

The following supporting information can be downloaded at https://www.mdpi.com/article/10.3390/curroncol30090615/s1, Supplementary methods; Supplementary safety; Supplementary Forest Diagrams and SUCRAs; Supplementary Subgroup Analysis; Supplementary Sensitivity Analysis; Supplementary Small-study Effects.

## Abbreviations

ABC	Advanced Breast Cancer
ADC	Antibody-Drug Conjugate
AEs	Adverse Events
AI	Aromatase Inhibitor
Akt	Protein Kinase B
CDK4/6	Cyclin-Dependent Kinase 4/6
CNS	Central Nervous System
CrI	Credible Interval
DIC	Error Information Criterion
EMA	European Medicines Agency
Endo	Endocrine Therapy
ER+	Estrogen Receptor Positive
ErbB	Human Epidermal Growth Factor Receptor
HER2+	Epidermal Growth Factor Receptor 2 Positive
Her2-ADC	Anti-Her2 Antibody-Drug Conjugate
Her2-mAb	Anti-Her2 Monoclonal Antibody
Her2-tki	Anti-Her2 Tyrosine Kinase Inhibitor
HR	Hazard Ratio
HRs	Hormone Receptors
mAb	Monoclonal Antibody
MBC	Metastatic Breast Cancer
mTOR	Mammalian Target of Rapamycin
NCCN	National Comprehensive Cancer Network
NMA	Network Meta-Analysis
OR	Odds Ratio
ORR	Objective Response Rate
OS	Overall Survival
p-Akt	Phosphorylated Akt
PD-1	Programmed Death 1
PD-L1	Programmed Death Ligand 1
PFS	Progression-Free Survival
PR+	Progesterone Receptor Positive
SUCRA	Surface Under the Cumulative Ranking Curve
T-DM1	Trastuzumab Emtansine
T-DXd	Trastuzumab Deruxtecan
TKI	Tyrosine Kinase Inhibitor

## References

[1] Sung H., Ferlay J., Siegel R.L., Laversanne M., Soerjomataram I., Jemal A., Bray F. (2021). Global Cancer Statistics 2020: GLOBOCAN Estimates of Incidence and Mortality Worldwide for 36 Cancers in 185 Countries. Ca-Cancer J. Clin..

[2] Dieci M.V., Guarneri V. (2020). Should triple-positive breast cancer be recognized as a distinct subtype?. Expert. Rev. Anticancer Ther..

[3] Gradishar W.J., Anderson B.O., Abraham J., Aft R., Agnese D., Allison K.H., Blair S.L., Burstein H.J., Dang C., Elias A.D. (2020). Breast Cancer, Version 3.2020, NCCN Clinical Practice Guidelines in Oncology. J. Natl. Compr. Cancer Netw..

[4] Kaufman B., Mackey J.R., Clemens M.R., Bapsy P.P., Vaid A., Wardley A., Tjulandin S., Jahn M., Lehle M., Feyereislova A. (2009). Trastuzumab Plus Anastrozole Versus Anastrozole Alone for the Treatment of Postmenopausal Women With Human Epidermal Growth Factor Receptor 2–Positive, Hormone Receptor–Positive Metastatic Breast Cancer: Results From the Randomized Phase III TAnDEM Study. J. Clin. Oncol..

[5] Schwartzberg L.S., Franco S.X., Florance A., O’Rourke L., Maltzman J., Johnston S. (2010). Lapatinib plus Letrozole as First-Line Therapy for HER-2+ Hormone Receptor–Positive Metastatic Breast Cancer. Oncologist.

[6] Huober J., Fasching P.A., Barsoum M., Petruzelka L., Wallwiener D., Thomssen C., Reimer T., Paepke S., Azim H.A., Ragosch V. (2012). Higher efficacy of letrozole in combination with trastuzumab compared to letrozole monotherapy as first-line treatment in patients with HER2-positive, hormone-receptor-positive metastatic breast cancer—Results of the eLEcTRA trial. Breast.

[7] Johnston S.R.D., Hegg R., Im S., Park I.H., Burdaeva O., Kurteva G., Press M.F., Tjulandin S., Iwata H., Simon S.D. (2021). Phase III, Randomized Study of Dual Human Epidermal Growth Factor Receptor 2 (HER2) Blockade With Lapatinib Plus Trastuzumab in Combination With an Aromatase Inhibitor in Postmenopausal Women With HER2-Positive, Hormone Receptor–Positive Metastatic Breast Cancer: Updated Results of ALTERNATIVE. J. Clin. Oncol..

[8] Rimawi M., Ferrero J.M., de la Haba-Rodriguez J., Poole C., De Placido S., Osborne C.K., Hegg R., Easton V., Wohlfarth C., Arpino G. (2018). First-Line Trastuzumab Plus an Aromatase Inhibitor, With or Without Pertuzumab, in Human Epidermal Growth Factor Receptor 2–Positive and Hormone Receptor–Positive Metastatic or Locally Advanced Breast Cancer (PERTAIN):A Randomized, Open-Label Phase II Trial. J. Clin. Oncol..

[9] Kay C., Martínez-Pérez C., Meehan J., Gray M., Webber V., Dixon J.M., Turnbull A.K. (2021). Current trends in the treatment of HR+/HER2+ breast cancer. Future Oncol..

[10] Feng F., Zhang T., Yin F., Liu C., Zhuang J., Qi L., Wang X., Li J., Wang L., Tian J. (2019). Efficacy and safety of targeted therapy for metastatic HER2-positive breast cancer in the first-line treatment: A Bayesian network meta-analysis. Oncotargets Ther..

[11] Zhang X., Leng J., Zhou Y., Mao F., Lin Y., Shen S., Sun Q. (2021). Efficacy and Safety of Anti-HER2 Agents in Combination With Chemotherapy for Metastatic HER2-Positive Breast Cancer Patient: A Network Meta-Analysis. Front. Oncol..

[12] Sadeghirad B., Foroutan F., Zoratti M.J., Busse J.W., Brignardello-Petersen R., Guyatt G., Thabane L. (2023). Theory and practice of Bayesian and frequentist frameworks for network meta-analysis. BMJ Evid.-Based Med..

[13] Shim S.R., Kim S.J., Lee J., Rucker G. (2019). Network meta-analysis: Application and practice using R software. Epidemiol. Health.

[14] Page M.J., McKenzie J.E., Bossuyt P.M., Boutron I., Hoffmann T.C., Mulrow C.D., Shamseer L., Tetzlaff J.M., Akl E.A., Brennan S.E. (2021). The PRISMA 2020 statement: An updated guideline for reporting systematic reviews. BMJ-Brit. Med. J..

[15] Hutton B., Salanti G., Caldwell D.M., Chaimani A., Schmid C.H., Cameron C., Ioannidis J.P., Straus S., Thorlund K., Jansen J.P. (2015). The PRISMA extension statement for reporting of systematic reviews incorporating network meta-analyses of health care interventions: Checklist and explanations. Ann. Intern. Med..

[16] Schulz K.F., Altman D.G., Moher D. (2010). CONSORT 2010 statement: Updated guidelines for reporting parallel group randomised trials. BMJ-Brit. Med. J..

[17] Andre F., Nadal J.C., Denys H., Goel S., Litchfield L.M., Appiah A., Chen Y., Tolaney S.M. (2022). LBA18 Final overall survival (OS) for abemaciclib plus trastuzumab +/− fulvestrant versus trastuzumab plus chemotherapy in patients with HR+, HER2+ advanced breast cancer (monarcHER): A randomized, open-label, phase II trial. Ann. Oncol..

[18] Takano T., Tsurutani J., Takahashi M., Yamanaka T., Sakai K., Ito Y., Fukuoka J., Kimura H., Kawabata H., Tamura K. (2018). A randomized phase II trial of trastuzumab plus capecitabine versus lapatinib plus capecitabine in patients with HER2-positive metastatic breast cancer previously treated with trastuzumab and taxanes: WJOG6110B/ELTOP. Breast.

[19] Awada A., Colomer R., Inoue K., Bondarenko I., Badwe R.A., Demetriou G., Lee S.C., Mehta A.O., Kim S.B., Bachelot T. (2016). Neratinib Plus Paclitaxel vs Trastuzumab Plus Paclitaxel in Previously Untreated Metastatic ERBB2-Positive Breast Cancer: The NEfERT-T Randomized Clinical Trial. JAMA Oncol..

[20] Harbeck N., Huang C.S., Hurvitz S., Yeh D.C., Shao Z., Im S.A., Jung K.H., Shen K., Ro J., Jassem J. (2016). Afatinib plus vinorelbine versus trastuzumab plus vinorelbine in patients with HER2-overexpressing metastatic breast cancer who had progressed on one previous trastuzumab treatment (LUX-Breast 1): An open-label, randomised, phase 3 trial. Lancet Oncol..

[21] Swain S.M., Baselga J., Kim S.B., Ro J., Semiglazov V., Campone M., Ciruelos E., Ferrero J.M., Schneeweiss A., Heeson S. (2015). Pertuzumab, trastuzumab, and docetaxel in HER2-positive metastatic breast cancer. N. Engl. J. Med..

[22] Andersson M., Lidbrink E., Bjerre K., Wist E., Enevoldsen K., Jensen A.B., Karlsson P., Tange U.B., Sørensen P.G., Møller S. (2011). Phase III Randomized Study Comparing Docetaxel Plus Trastuzumab With Vinorelbine Plus Trastuzumab As First-Line Therapy of Metastatic or Locally Advanced Human Epidermal Growth Factor Receptor 2–Positive Breast Cancer: The HERNATA Study. J. Clin. Oncol..

[23] Baselga J., Manikhas A., Cortés J., Llombart A., Roman L., Semiglazov V.F., Byakhov M., Lokanatha D., Forenza S., Goldfarb R.H. (2014). Phase III trial of nonpegylated liposomal doxorubicin in combination with trastuzumab and paclitaxel in HER2-positive metastatic breast cancer. Ann. Oncol..

[24] Fei Ma M., Quchang Ouyang M., Wei Li M., Zefei Jiang M., Zhongsheng Tong M., Yunjiang Liu M., Huiping Li M.P., Shiying Yu M., Jifeng Feng M., Shusen Wang M. (2019). Pyrotinib or Lapatinib Combined With Capecitabine in HER2–Positive Metastatic Breast Cancer With Prior Taxanes, Anthracyclines, and/or Trastuzumab: A Randomized, Phase II Study. J. Clin. Oncol..

[25] Murthy R.K., Loi S., Okines A., Paplomata E., Hamilton E., Hurvitz S.A., Lin N.U., Borges V., Abramson V., Anders C. (2020). Tucatinib, Trastuzumab, and Capecitabine for HER2-Positive Metastatic Breast Cancer. N. Engl. J. Med..

[26] Sim S.H., Park I.H., Jung K.H., Kim S., Ahn J., Lee K., Im S., Im Y., Park Y.H., Sohn J. (2019). Randomised Phase 2 study of lapatinib and vinorelbine vs vinorelbine in patients with HER2 +  metastatic breast cancer after lapatinib and trastuzumab treatment (KCSG BR11-16). Brit. J. Cancer.

[27] von Minckwitz G., Schwedler K., Schmidt M., Barinoff J., Mundhenke C., Cufer T., Maartense E., de Jongh F.E., Baumann K.H., Bischoff J. (2011). Trastuzumab beyond progression: Overall survival analysis of the GBG 26/BIG 3-05 phase III study in HER2-positive breast cancer. Eur. J. Cancer.

[28] Sunil Verma M.D.D.M. (2012). Trastuzumab Emtansine for HER2-Positive Advanced Breast Cancer. N. Engl. J. Med..

[29] Emens L.A., Esteva F.J., Beresford M., Saura C., De Laurentiis M., Kim S.B., Im S.A., Wang Y., Salgado R., Mani A. (2020). Trastuzumab emtansine plus atezolizumab versus trastuzumab emtansine plus placebo in previously treated, HER2-positive advanced breast cancer (KATE2): A phase 2, multicentre, randomised, double-blind trial. Lancet Oncol..

[30] Perez E.A., Barrios C., Eiermann W., Toi M., Im Y., Conte P., Martin M., Pienkowski T., Pivot X., Burris H.A. (2017). Trastuzumab Emtansine With or Without Pertuzumab Versus Trastuzumab Plus Taxane for Human Epidermal Growth Factor Receptor 2–Positive, Advanced Breast Cancer: Primary Results From the Phase III MARIANNE Study. J. Clin. Oncol..

[31] Xu B., Yan M., Ma F., Hu X., Feng J., Ouyang Q., Tong Z., Li H., Zhang Q., Sun T. Updated overall survival (OS) results from the phase 3 PHOEBE trial of pyrotinib versus la patinib in combination with capecitabine in patients with HER2-positive metastatic breast cancer. Proceedings of the San Antonio Breast Cancer Symposium (SABCS).

[32] Ciruelos E., Villagrasa P., Pascual T., Oliveira M., Pernas S., Paré L., Escrivá-de-Romaní S., Manso L., Adamo B., Martínez E. (2020). Palbociclib and Trastuzumab in HER2-Positive Advanced Breast Cancer: Results from the Phase II SOLTI-1303 PATRICIA Trial. Clin. Cancer Res..

[33] Cortés J., Kim S.B., Chung W.P., Im S.A., Park Y.H., Hegg R., Kim M.H., Tseng L.M., Petry V., Chung C.F. (2022). Trastuzumab Deruxtecan versus Trastuzumab Emtansine for Breast Cancer. N. Engl. J. Med..

[34] Yamamoto Y., Iwata H., Taira N., Masuda N., Takahashi M., Yoshinami T., Ueno T., Toyama T., Yamanaka T., Takano T. (2022). Pertuzumab retreatment for HER2-positive advanced breast cancer: A randomized, open-label phase III study (PRECIOUS). Cancer Sci..

[35] Saura C., Oliveira M., Feng Y.H., Dai M.S., Chen S.W., Hurvitz S.A., Kim S.B., Moy B., Delaloge S., Gradishar W. (2020). Neratinib Plus Capecitabine Versus Lapatinib Plus Capecitabine in HER2-Positive Metastatic Breast Cancer Previously Treated With >/= 2 HER2-Directed Regimens: Phase III NALA Trial. J. Clin. Oncol..

[36] André F., O’Regan R., Ozguroglu M., Toi M., Xu B., Jerusalem G., Masuda N., Wilks S., Arena F., Isaacs C. (2014). Everolimus for women with trastuzumab-resistant, HER2-positive, advanced breast cancer (BOLERO-3): A randomised, double-blind, placebo-controlled phase 3 trial. Lancet Oncol..

[37] Scheuer W., Friess T., Burtscher H., Bossenmaier B., Endl J., Hasmann M. (2009). Strongly enhanced antitumor activity of trastuzumab and pertuzumab combination treatment on HER2-positive human xenograft tumor models. Cancer Res..

[38] Nahta R., Hung M.C., Esteva F.J. (2004). The HER-2-targeting antibodies trastuzumab and pertuzumab synergistically inhibit the survival of breast cancer cells. Cancer Res..

[39] Swain S.M., Miles D., Kim S.B., Im Y.H., Im S.A., Semiglazov V., Ciruelos E., Schneeweiss A., Loi S., Monturus E. (2020). Pertuzumab, trastuzumab, and docetaxel for HER2-positive metastatic breast cancer (CLEOPATRA): End-of-study results from a double-blind, randomised, placebo-controlled, phase 3 study. Lancet Oncol..

[40] Miles D., Ciruelos E., Schneeweiss A., Puglisi F., Peretz-Yablonski T., Campone M., Bondarenko I., Nowecki Z., Errihani H., Paluch-Shimon S. (2021). Final results from the PERUSE study of first-line pertuzumab plus trastuzumab plus a taxane for HER2-positive locally recurrent or metastatic breast cancer, with a multivariable approach to guide prognostication. Ann. Oncol..

[41] Xia W., Gerard C.M., Liu L., Baudson N.M., Ory T.L., Spector N.L. (2005). Combining lapatinib (GW572016), a small molecule inhibitor of ErbB1 and ErbB2 tyrosine kinases, with therapeutic anti-ErbB2 antibodies enhances apoptosis of ErbB2-overexpressing breast cancer cells. Oncogene.

[42] Giampaglia M., Chiuri V.E., Tinelli A., De Laurentiis M., Silvestris N., Lorusso V. (2010). Lapatinib in breast cancer: Clinical experiences and future perspectives. Cancer Treat. Rev..

[43] Arteaga C.L., Sliwkowski M.X., Osborne C.K., Perez E.A., Puglisi F., Gianni L. (2011). Treatment of HER2-positive breast cancer: Current status and future perspectives. Nat. Rev. Clin. Oncol..

[44] Freedman R.A., Gelman R.S., Anders C.K., Melisko M.E., Parsons H.A., Cropp A.M., Silvestri K., Cotter C.M., Componeschi K.P., Marte J.M. (2019). TBCRC 022: A Phase II Trial of Neratinib and Capecitabine for Patients With Human Epidermal Growth Factor Receptor 2-Positive Breast Cancer and Brain Metastases. J. Clin. Oncol..

[45] Ferraro E., Drago J.Z., Modi S. (2021). Implementing antibody-drug conjugates (ADCs) in HER2-positive breast cancer: State of the art and future directions. Breast Cancer Res..

