# Supplementary material for: Efficacy and Safety of Anti-HER2 Targeted Therapy for Metastatic HR-Positive and HER2-Positive Breast Cancer: A Bayesian Network Meta-Analysis
Source: Curr Oncol. 2023 Sep 15;30(9):8444–63. doi: 10.3390/curroncol30090615 (PMC10528081; doi:10.3390/curroncol30090615)
Supplement: Supplementary file 1 [file curroncol-30-00615-s001.zip › curroncol-2535353-supplementary.pdf]

# Supplementary appendix

**Supplement to:**

## Efficacy and safety of anti-HER2 targeted therapy for metastatic HR-positive and HER2-positive breast cancer: A Bayesian Network Meta-Analysis

Xian-Meng Wu[1], Yong-Kang Qian[1], Hua-Ling Chen[1], Chen-Hua Hu[1], Bing-Wei Chen[1*]

[1] Department of Epidemiology and Health Statistics, School of Public Health, Southeast University, Nanjing 210009, P.R. China.

*** Correspondence:**

BingWei Chen

drchenbw@126.com

# content


**Supplementary Methods** ........ 4

**Table S1.** Search strategy ........ 4

**Supplementary Safety** ........ 13

**Table S2.** Random-effects model Bayesian network meta-analysis for estimating ORs for indicators of the incidence of grade 3/4 adverse events among different treatment regimens ........ 13

**Supplementary Forest Diagrams and SUCRAs** ........ 14

**Figure S1.** The forest plot for the PFS#1 after meta-analysis of the random-effects model Bayesian network ........ 14

**Figure S2.** The SUCRA composite ranking rank of treatment regimens after Bayesian network meta-analysis of the random effects model for PFS#1 ........ 15

**Figure S3.** The forest plot for the PFS#2 after meta-analysis of the random-effects model Bayesian network ........ 16

**Figure S4.** Random-effects model Bayesian network meta-analysis for PFS#2 after treatment regimen SUCRA integrated ranking scale ........ 17

**Figure S5.** The forest plot for the OS#1 after meta-analysis of the random-effects model Bayesian network ........ 18

**Figure S6.** Random-effects model Bayesian network meta-analysis for OS#1 with the SUCRA composite ranking rank of treatment options ........ 19

**Figure S7.** The forest plot for the OS#2 after meta-analysis of the random-effects model Bayesian network ........ 20

**Figure S8.** Random-effects model Bayesian network meta-analysis for OS#2 after SUCRA combined ranking ranking of treatment regimens ........ 21

**Figure S9.** The forest plot for the ORR#1 after meta-analysis of the random-effects model Bayesian network ........ 22

**Figure S10.** Random-effects model Bayesian network meta-analysis for ORR#1 with the SUCRA composite ranking rank of treatment options ........ 23

**Figure S11.** The forest plot for the ORR#2 after meta-analysis of the random-effects model Bayesian network ........ 24

**Figure S12.** Random-effects model Bayesian network meta-analysis for ORR#2 with SUCRA composite ranking of treatment options ........ 25

**Figure S13.** The forest plot for the grade 3/4 adverse events after meta-analysis of the random-effects model Bayesian network ........ 26

**Figure S14.** Random-effects model Bayesian network meta-analysis of treatment regimens after SUCRA composite ranking scale for grade 3/4 adverse events ........ 27

**Supplementary Subgroup Analysis** ........ 28

**Table S3.** Bayesian network meta-analysis of PFS#2 estimated HR between different treatment regimens (number of treatment lines equal to 1) ........ 28

**Table S4.** Bayesian network meta-analysis of PFS#2 estimated HR between different treatment regimens (number of treatment lines >1) ........ 29

**Supplementary Sensitivity Analysis** ........ 30

**Table S5.** Fixed effects model Bayesian network meta-analysis of ORR#1 between different treatment regimens Estimated OR ........ 30
**Table S6.** Fixed effects model Bayesian network meta-analysis of ORR#2 between different treatment regimens to estimate OR ........ 30
**Table S7.** Fixed-effects model Bayesian network meta-analysis of PFS#1 estimated HRs between different treatment regimens ........ 31
**Table S8.** Fixed-effects model Bayesian network meta-analysis of PFS#2 estimated ORs between different treatment regimens ........ 32
**Table S9.** Fixed effects model Bayesian network meta-analysis of OS#1 estimated ORs between different treatment regimens ........ 33
**Table S10.** Fixed-effects model Bayesian network meta-analysis of OS#2 estimated ORs between different treatment regimens ........ 33
**Table S11.** Fixed-effects model Bayesian network meta-analysis of the incidence of grade 3/4 adverse events between different treatment regimens to estimate OR ........ 34
**Supplementary Small-study Effects** ........ 35
**Figure S15.** Comparative-corrected funnel plots for ORR#1 ........ 35
**Figure S16.** Comparative-corrected funnel plots for ORR#2 ........ 36
**Figure S17.** Comparative-corrected funnel plots for PFS#1 ........ 37
**Figure S18.** Comparative-corrected funnel plots for PFS#2 ........ 38
**Figure S19.** Comparative-corrected funnel plots for OS#1 ........ 39
**Figure S20.** Comparative-corrected funnel plots for OS#2 ........ 40
**Figure S21.** Comparative-corrected funnel plots for grade 3/4 adverse events ........ 41

# Supplementary Methods

**Table S1**. Search strategy

| Database | Retrieval strategy |
|---|---|
| CNKI database | **Chinese：**((SU="乳腺肿瘤") OR (TKA="乳腺肿瘤，人类") OR (TKA="乳腺癌") OR (TKA="乳腺癌症") OR (TKA="乳腺瘤") OR (TKA="人类乳腺肿瘤")) and ((SU="受体, erbB-2") OR (TKA="HER2+") OR (TKA="HER2 阳性") OR (TKA="表皮生长因子受体 2 阳性")) and (((TKA="HR 阳性") OR (TKA="HR+") OR (TKA="激素受体阳性")) or ((SU="受体，雄激素") OR (TKA="PR 阳性") OR (TKA="PR+") OR (TKA="雄激素受体阳性")) or ((SU="受体，雌激素") OR (TKA="ER 阳性") OR (TKA="ER+") OR (TKA="雌激素受体阳性"))) and ((SU="随机对照试验") OR (TKA="对照临床试验，随机") OR (TKA="临床试验"))<br><br>**English：**((SU="Breast Tumor") OR (TKA="Breast Tumor, Human") OR (TKA="Breast Cancer") OR (TKA="Breast Carcinoma") OR (TKA="Breast Neoplasm") OR (TKA="Human Breast Tumor")) and ((SU="Receptor, erbB-2") OR (TKA="HER2+") OR (TKA="HER2 Positive") OR (TKA="Epidermal Growth Factor Receptor 2 Positive")) and (((TKA="HR+") OR (TKA="HR Positive") OR (TKA="Hormone Receptor Positive")) or ((SU="Receptor, Androgen") OR (TKA="PR+") OR (TKA="PR Positive") OR (TKA="Androgen Receptor Positive")) or ((SU="Receptor, Estrogen") OR (TKA="ER+") OR (TKA="ER Positive") OR (TKA="Estrogen Receptor Positive"))) and ((SU="Randomized Controlled Trial") OR (TKA="Randomized Clinical Study") OR (TKA="Clinical Trial, Randomized") OR (TKA="Clinical Trial")) |
| Wan Fang database | **Chinese：**(主题:（“乳腺肿瘤") OR 题名或关键词: ( "乳腺肿瘤，人类" OR "乳腺癌" OR "乳腺癌症" OR "乳腺瘤" OR "人类乳腺肿瘤")) and (主题:("受体, erbB-2") OR 题名或关键词: ("HER2+" OR "HER2 阳性" OR "表皮生长因子受体 2 阳性")) and (题名或关键词: ("HR 阳性" OR "HR+" OR "激素受体阳性") or (主题:("受体，雄激素") OR (题名或关键词: ("PR 阳性" OR "PR+" OR "雄激素受体阳性")) or (主题:("受体，雌激素") OR 题名或关键词: ("ER 阳性" OR "ER+" OR "雌激素受体阳性))) and (主题:("随机对照试验") OR 题名或关键词:("对照临床试验，随机" OR "临床试验，随机" OR "临床研究"))<br><br>**English：**(Text word: "Breast Tumor" OR Title/Keywords: ("Breast Tumor, Human" OR "Breast Cancer" OR "Breast Carcinoma" OR "Breast |

| | |
|---|---|
| | Neoplasm" OR "Human Breast Tumor")) and (Text word: "Receptor, erbB-2" OR Title/Keywords: ("HER2 Positive" OR "HER2 Positive" OR "Epidermal Growth Factor Receptor 2 Positive")) and (Title/Keywords: ("HR Positive" OR "HR Positive" OR "Hormone Receptor Positive") or (Text word: "Receptor, Androgen" OR Title/Keywords: ("PR Positive" OR "PR Positive" OR "Androgen Receptor Positive")) or (Text word: "Receptor, Estrogen" OR Title/Keywords: ("ER Positive" OR "ER Positive" OR "Estrogen Receptor Positive"))) and (Text word: "Randomized Controlled Trial" OR Title/Keywords: ("Randomized Clinical Study" OR "Clinical Trial, Randomized" OR "Clinical Research")) |
| Sinomed database | **Chinese：**<br>序号 检索表达式 命中文献数 检索时间<br>2) "受体, erbB-2"[不加权:扩展] 3637 2022-11-24 16:14:41.0<br>3) "HER2+"[常用字段:智能] OR "HER2 阳性"[常用字段:智能] OR "表皮生长因子受体 2 阳性"[常用字段:智能] 3960 2022-11-24 16:15:32.0<br>4) "受体, 雌激素"[不加权:扩展] 11468 2022-11-24 16:16:11.0<br>5) "ER 阳性"[常用字段:智能] OR "ER+"[常用字段:智能] OR "雌激素受体阳性"[常用字段:智能] 16123 2022-11-24 16:16:44.0<br>6) "受体, 雄激素"[不加权:扩展] 5912 2022-11-24 16:17:09.0<br>7) "PR 阳性"[常用字段:智能] OR "PR+"[常用字段:智能] OR "雄激素受体阳性"[常用字段:智能] 31373 2022-11-24 16:17:41.0<br>8) "HR 阳性"[常用字段:智能] OR "HR+"[常用字段:智能] OR "激素受体阳性"[常用字段:智能] 66274 2022-11-24 16:18:09.0<br>9) "随机对照试验"[不加权:扩展] 199665 2022-11-24 16:18:38.0<br>10) "对照临床试验,"[常用字段:智能] AND "随机"[常用字段:智能] OR "临床试验,"[常用字段:智能] AND "随机"[常用字段:智能] OR "临床研究"[常用字段:智能] 431901 2022-11-24 16:19:33.0<br>18) ("乳腺肿瘤"[不加权:扩展]) AND ("2022"[时间] OR "2021"[时间] OR "2020"[时间] OR "2019"[时间] OR "2018"[时间]) 26016 2022-11-24 16:43:05.0 |

| | |
|---|---|
| | 19) "乳腺肿瘤,"[常用字段:智能] AND "人类"[常用字段:智能] OR "乳腺癌"[常用字段:智能] OR "乳腺癌症"[常用字段:智能] OR "乳腺瘤"[常用字段:智能] OR "人类乳腺肿瘤"[常用字段:智能] 128229 2022-11-24 16:50:01.0<br>20) (#19) OR (#18) 128229 2022-11-24 16:52:09.0<br>21) (#3) OR (#2) 7408 2022-11-24 16:52:28.0<br>22) (#8) OR (#7) OR (#6) OR (#5) OR (#4) 113774 2022-11-24 16:52:54.0<br>23) (#10) OR (#9) 431901 2022-11-24 16:53:11.0<br>24) (#23) AND (#22) AND (#21) AND (#20) 77 2022-11-24 16:53:51.0<br><br>**English:**<br>Number Expression Retrieved Documents Retrieval Date:<br>2) "Receptor, erbB-2"[Unweighted: Expanded] 3637 2022-11-24 16:14:41.0<br>3) "HER2+"[Common Fields: Intelligent] OR "HER2 positive"[Common Fields: Intelligent] OR "Human epidermal growth factor receptor 2 positive"[Common Fields: Intelligent] 3960 2022-11-24 16:15:32.0<br>4) " Receptor, Estrogen"[Unweighted: Expanded] 11468 2022-11-24 16:16:11.0<br>5) "ER positive"[Common Fields: Intelligent] OR "ER+"[Common Fields: Intelligent] OR "Receptors, Estrogen positive"[Common Fields: Intelligent] 16123 2022-11-24 16:16:44.0<br>6) "Receptor, Androgen"[Unweighted: Expanded] 5912 2022-11-24 16:17:09.0<br>7) "PR positive"[Common Fields: Intelligent] OR "PR+"[Common Fields: Intelligent] OR "Androgen Receptor positive"[Common Fields: Intelligent] 31373 2022-11-24 16:17:41.0<br>8) "HR positive"[Common Fields: Intelligent] OR "HR+"[Common Fields: Intelligent] OR "Hormone Receptor positive"[Common Fields: Intelligent] 66274 2022-11-24 16:18:09.0<br>9) "Randomized Controlled Trial"[Unweighted: Expanded] 199665 2022-11-24 16:18:38.0 |

| | |
|---|---|
| | 10) "Controlled Trial,"[Common Fields: Intelligent] AND "Randomized"[Common Fields: Intelligent] OR "Clinial Trial"[Common Fields: Intelligent] AND "random"[Common Fields: Intelligent] OR "Clinial Study"[Common Fields: Intelligent] 431901 2022-11-24 16:19:33.0<br>18) ("Breast Neoplasms"[Unweighted: Expanded]) AND ("2022"[Time] OR "2021"[Time] OR "2020"[Time] OR "2019"[Time] OR "2018"[Time]) 26016 2022-11-24 16:43:05.0<br>19) "Breast Neoplasms,"[Common Fields: Intelligent] AND "Human"[Common Fields: Intelligent] OR "Breast Neoplasms"[Common Fields: Intelligent] OR "Breast Tumors"[Common Fields: Intelligent] OR "Malignant Tumor of Breast"[Common Fields: Intelligent] OR "Human Mammary Carcinoma"[Common Fields: Intelligent] 128229 2022-11-24 16:50:01.0<br>20) (#19) OR (#18) 128229 2022-11-24 16:52:09.0<br>21) (#3) OR (#2) 7408 2022-11-24 16:52:28.0<br>22) (#8) OR (#7) OR (#6) OR (#5) OR (#4) 113774 2022-11-24 16:52:54.0<br>23) (#10) OR (#9) 431901 2022-11-24 16:53:11.0<br>24) (#23) AND (#22) AND (#21) AND (#20) 77 2022-11-24 16:53:51.0 |
| PubMed database | ((("Breast Neoplasms"[Mesh]) AND ((((((((((((((((((((( (((((((((((((Breast Neoplasms[Title/Abstract]) OR (Neoplasm, Breast[Title/Abstract])) OR (Neoplasms, Breast[Title/Abstract])) OR (Breast Tumors[Title/Abstract])) OR (Breast Tumor[Title/Abstract])) OR (Tumor, Breast[Title/Abstract])) OR (Tumors, Breast[Title/Abstract])) OR (Breast Cancer[Title/Abstract])) OR (Cancer, Breast[Title/Abstract])) OR (Malignant Tumor of Breast[Title/Abstract])) OR (Breast Malignant Tumor[Title/Abstract])) OR (Breast Malignant Tumors[Title/Abstract])) OR (Cancer of the Breast[Title/Abstract])) OR (Cancer of Breast[Title/Abstract])) OR (Malignant Neoplasm of Breast[Title/Abstract])) OR (Breast Malignant Neoplasm)) OR (Breast Malignant Neoplasms)) OR (Mammary Cancer)) OR (Cancer, Mammary)) OR (Cancers, Mammary)) OR (Mammary Cancers)) OR (Mammary arcinoma, Human)) OR (Carcinoma, Human Mammary)) OR (Carcinomas, Human Mammary)) OR (Human Mammary Carcinomas)) OR (Mammary Carcinomas, Human)) OR (Human Mammary Carcinoma)) OR (Mammary Neoplasms, Human)) OR (Human Mammary Neoplasm)) OR (Human Mammary Neoplasms)) OR (Neoplasm, Human |

| | |
|---|---|
| | Mammary)) OR (Neoplasms, Human Mammary)) OR (Mammary Neoplasm, Human)) OR (Breast Carcinoma)) OR (Breast Carcinomas)) OR (Carcinoma, Breast)) OR (Carcinomas, Breast))) AND (((((((((((((HER2+[Title/Abstract]) OR (HER2 positive[Title/Abstract])) OR (HER2+ human epidermal growth factor receptor 2 positive[Title/Abstract])) OR (PR+[Title/Abstract])) OR (Receptors, Progesterone positive[Title/Abstract])) OR (Progesterone receptor positive[Title/Abstract])) OR (ER+[Title/Abstract])) OR (Receptors, Estrogen positive[Title/Abstract])) OR (Estrogen Receptors positive[Title/Abstract])) OR (HR+[Title/Abstract])) OR (HR positive[Title/Abstract])) OR (Hormone receptor positive[Title/Abstract])) OR (TPBC[Title/Abstract])) OR (Triple positive breast cancer[Title/Abstract]))) AND (((((((((((Randomized controlled trial[Title/Abstract]) OR (controlled clinical trial[Title/Abstract])) OR (random allocation[Title/Abstract])) OR (double-blind[Title/Abstract])) OR (single-blind[Title/Abstract])) OR (placebo[Title/Abstract])) OR (randomly[Title/Abstract])) OR (randomized[Title/Abstract])) OR (clinical trial[Title/Abstract])) OR (trial[Title/Abstract])) OR (RCT[Title/Abstract])) OR (random[Title/Abstract])) |
| Embase database | No. Query Results Results Date<br>#7. #1 AND #2 AND #3 AND #4 AND #5 AND #6 AND ([chinese]/lim OR [english]/lim) AND [2000-2022]/py AND ([article]/lim OR [article in press]/lim OR [review]/lim OR [short survey]/lim) AND [humans]/lim 1,370 23 Nov 2022<br>#6. 'randomize control trial' OR (randomize AND ('control'/exp OR control) AND ('trial'/exp OR trial)) OR 'controlled clinical trial'/exp OR 'controlled clinical trial' OR (controlled AND ('clinical'/exp OR clinical) AND ('trial'/exp OR trial)) OR 'random allocation'/exp OR 'random 3,639,808 23 Nov 2022 |

| | | | |
|---|---|---|---|
| | allocation' OR (random AND allocation) OR 'double blind' OR 'single blind' OR 'placebo'/exp OR placebo OR randomly OR randomized OR 'clinical trial'/exp OR 'clinical trial' OR (('clinical'/exp OR clinical) AND ('trial'/exp OR trial)) OR 'trial'/exp OR trial OR rct OR random | | |
| | #5. 'controlled study'/exp | 9,447,635 | 23 Nov 2022 |
| | #4. pr+ OR 'receptors, progesterone positive' OR (receptors, AND ('progesterone'/exp OR progesterone) AND positive) OR 'progesterone receptor positive' OR (('progesterone'/exp OR progesterone) AND ('receptor'/exp OR receptor) AND positive) OR 'er+'/exp OR er+ OR 'receptors, estrogen positive' OR (receptors, AND ('estrogen'/exp OR estrogen) AND positive) OR 'estrogen receptors positive' OR (('estrogen'/exp OR estrogen) AND ('receptors'/exp OR receptors) AND positive) OR hr+ OR 'hr positive' OR (hr AND positive) OR 'hormone receptor positvie' OR (('hormone'/exp OR hormone) AND ('receptor'/exp OR receptor) AND positvie) OR tpbc OR 'triple positive breast cancer'/exp OR 'triple positive | 954,122 | 23 Nov 2022 |

| | | | |
|---|---|---|---|
| | breast cancer' OR (triple AND positive AND ('breast'/exp OR breast) AND ('cancer'/exp OR cancer)) | | |
| | #3. her2+:ab,ti OR 'her2 positvie':ab,ti OR 'human epidermal growth factor receptor 2 positvie':ab,ti | 76,134 | 23 Nov 2022 |
| | #2. 'neoplasm, breast':ab,ti OR 'neoplasms, breast':ab,ti OR 'breast tumors':ab,ti OR 'breast tumor':ab,ti OR 'tumor, breast':ab,ti OR 'tumors, breast':ab,ti OR 'breast cancer':ab,ti OR 'cancer, breast':ab,ti OR 'malignant tumor of breast':ab,ti OR 'breast malignant tumor':ab,ti OR 'breast malignant tumors':ab,ti OR 'cancer of the breast':ab,ti OR 'cancer of breast':ab,ti OR 'malignant neoplasm of breast':ab,ti OR 'breast malignant neoplasm':ab,ti OR 'breast malignant neoplasms':ab,ti OR 'mammary cancer':ab,ti OR 'cancer, mammary':ab,ti OR 'cancers, mammary':ab,ti OR 'mammary cancers':ab,ti OR 'mammary arcinoma, human':ab,ti OR 'carcinoma, human mammary':ab,ti OR 'carcinomas, human mammary':ab,ti OR 'human mammary | 499,726 | 23 Nov 2022 |

| | |
|---|---|
| | carcinomas':ab,ti OR 'mammary carcinomas, human':ab,ti OR 'human mammary carcinoma':ab,ti OR 'mammary neoplasms, human':ab,ti OR 'human mammary neoplasm':ab,ti OR 'human mammary neoplasms':ab,ti OR 'neoplasm, human mammary':ab,ti OR 'neoplasms, human mammary':ab,ti OR 'mammary neoplasm, human':ab,ti OR 'breast carcinoma':ab,ti OR 'breast carcinomas':ab,ti OR 'carcinoma, breast':ab,ti OR 'carcinomas, breast':ab,ti<br>#1. 'breast tumor'/exp 646,700 23 Nov 2022 |
| The Cochrane Library database | #1: MeSH descriptor: [Breast Neoplasms] explode all trees<br>#2: (Neoplasm, Breast OR Neoplasms, Breast OR Breast Tumors OR Breast Tumor OR Tumor, Breast OR Tumors, Breast OR Breast Cancer OR Cancer, Breast OR Malignant Tumor of Breast OR Breast Malignant Tumor OR Breast Malignant Tumors OR Cancer of the Breast OR Cancer of Breast OR Malignant Neoplasm of Breast OR Breast Malignant Neoplasm OR Breast Malignant Neoplasms OR Mammary Cancer OR Cancer, Mammary OR Cancers, Mammary OR Mammary Cancers OR Mammary arcinoma, Human OR Carcinoma, Human Mammary OR Carcinomas, Human Mammary OR Human Mammary Carcinomas OR Mammary Carcinomas, Human OR Human Mammary Carcinoma OR Mammary Neoplasms, Human OR Human Mammary Neoplasm OR Human Mammary Neoplasms OR Neoplasm, Human Mammary OR Neoplasms, Human Mammary OR Mammary Neoplasm, Human OR Breast Carcinoma OR Breast Carcinomas OR Carcinoma, Breast OR Carcinomas, Breast):ti,ab,kw<br>#3: #1 or #2<br>#4: (HER2 OR HER2 positive OR Human epidermal growth factor receptor 2 positive):ti,ab,kw<br>#5: (PR OR Receptors, Progesterone positive OR Progesterone receptor positive OR ER OR Receptors, Estrogen positive OR Estrogen Receptors positive OR HR OR HR positive OR Hormone receptor positive OR TPBC or Triple positive breast cancer):ti,ab,kw |

| | |
|---|---|
| | #6: MeSH descriptor: [Randomized Controlled Trial] explode all trees<br>#7: (controlled clinical trial OR random allocation OR double-blind OR single-blind OR placebo OR randomly OR randomized OR clinical trial OR trial OR RCT OR random):ti,ab,kw<br>#8: #6 or #7<br>#9: #3 and #4 and #5 and #8 |
| Web of Science database | (TS=(Breast Neoplasms) OR AB=(Neoplasm, Breast OR Neoplasms, Breast OR Breast Tumors OR Breast Tumor OR Tumor, Breast OR Tumors, Breast OR Breast Cancer OR Cancer, Breast OR Malignant Tumor of Breast OR Breast Malignant Tumor OR Breast Malignant Tumors OR Cancer of the Breast OR Cancer of Breast OR Malignant Neoplasm of Breast OR Breast Malignant Neoplasm OR Breast Malignant Neoplasms OR Mammary Cancer OR Cancer, Mammary OR Cancers, Mammary OR Mammary Cancers OR Mammary arcinoma, Human OR Carcinoma, Human Mammary OR Carcinomas, Human Mammary OR Human Mammary Carcinomas OR Mammary Carcinomas, Human OR Human Mammary Carcinoma OR Mammary Neoplasms, Human OR Human Mammary Neoplasm OR Human Mammary Neoplasms OR Neoplasm, Human Mammary OR Neoplasms, Human Mammary OR Mammary Neoplasm, Human OR Breast Carcinoma OR Breast Carcinomas OR Carcinoma, Breast OR Carcinomas, Breast)) AND AB=(HER2+ OR HER2 positive OR Human epidermal growth factor receptor 2 positive) AND AB=(PR+ OR Receptors, Progesterone positive OR Progesterone receptor positive OR ER+ OR Receptors, Estrogen positive OR Estrogen Receptors positive OR HR+ OR HR positive OR Hormone receptor positive OR TPBC or Triple positive breast cancer) AND (TS=(Randomized controlled trial) OR AB=(controlled clinical trial OR random allocation OR double-blind OR single-blind OR placebo OR randomly OR randomized OR clinical trial OR trial OR RCT OR random)) |

## Supplementary Safety

**Table S2.** Random-effects model Bayesian network meta-analysis for estimating ORs for indicators of the incidence of grade 3/4 adverse events among different treatment regimens

| | | | | | | | | |
|---|---|---|---|---|---|---|---|---|
| **Her2-mAb+Chem** | 2.28 (0.38, 14.91) | 1.07 (0.17, 6.77) | 0.4 (0.06, 2.94) | 0.42 (0.06, 3.03) | 2.17 (0.32, 15.22) | 0.73 (0.05, 11.31) | 0.66 (0.04, 10.24) | 0.05 (0, 1.65) |
| 0.44 (0.07, 2.66) | **Her2-mAb+CDK4/6+Endo** | 0.47 (0.1, 2.07) | 0.18 (0.01, 2.56) | 0.18 (0.01, 2.66) | 0.95 (0.06, 13.67) | 0.32 (0.01, 8.44) | 0.29 (0.01, 7.76) | 0.02 (0, 1.07) |
| 0.93 (0.15, 5.86) | 2.13 (0.48, 10.06) | **Her2-mAb+CDK4/6** | 0.38 (0.03, 5.5) | 0.39 (0.03, 5.7) | 2.04 (0.14, 29.98) | 0.67 (0.03, 17.73) | 0.62 (0.02, 16.49) | 0.05 (0, 2.32) |
| 2.5 (0.34, 17.28) | 5.69 (0.39, 82.43) | 2.66 (0.18, 37.23) | **Her2-mAb+Endo** | 1.05 (0.14, 7.81) | 5.41 (0.76, 38.44) | 1.79 (0.26, 12.11) | 1.64 (0.24, 11.01) | 0.13 (0, 2.24) |
| 2.37 (0.33, 16.65) | 5.43 (0.38, 78.31) | 2.54 (0.18, 35.81) | 0.95 (0.13, 7.01) | **Her2-mAb+Her2-mAb+Endo** | 5.12 (0.73, 37.06) | 1.72 (0.11, 27.34) | 1.56 (0.1, 24.64) | 0.12 (0, 3.95) |
| 0.46 (0.07, 3.14) | 1.05 (0.07, 15.5) | 0.49 (0.03, 6.91) | 0.18 (0.03, 1.31) | 0.2 (0.03, 1.37) | **Her2-mAb+Her2-mAb+Chem** | 0.33 (0.02, 5.2) | 0.3 (0.02, 4.52) | **0.02 (0, 0.75)** |
| 1.38 (0.09, 21.06) | 3.15 (0.12, 83.25) | 1.49 (0.06, 38.55) | 0.56 (0.08, 3.78) | 0.58 (0.04, 9.29) | 3 (0.19, 46.84) | **Her2-mAb+Her2-tki+Endo** | 0.91 (0.14, 6.13) | 0.07 (0, 2.18) |
| 1.51 (0.1, 22.81) | 3.44 (0.13, 93.79) | 1.61 (0.06, 42.59) | 0.61 (0.09, 4.15) | 0.64 (0.04, 10.09) | 3.31 (0.22, 51.15) | 1.1 (0.16, 7.13) | **Her2-tki+Endo** | 0.08 (0, 2.41) |
| 19.48 (0.6, 1397.14) | 45.07 (0.93, 4683.6) | 21.15 (0.43, 2121.1) | 7.65 (0.45, 372.21) | 8.24 (0.25, 611.17) | **42.45 (1.33, 3035.49)** | 13.9 (0.46, 1042.41) | 12.62 (0.42, 971.93) | **Endo** |

## Supplementary Forest Diagrams and SUCRAs

**Figure S1.** The forest plot for the PFS[#1] after meta-analysis of the random-effects model Bayesian network

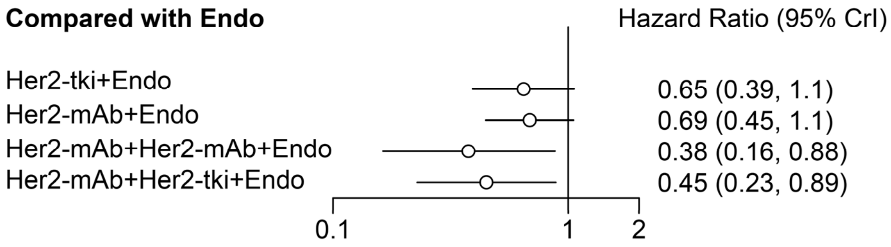


**Legend.** When HR and 95% CrI are less than 1, it indicates that the anti-HER2 combined with endocrine therapy has significant benefit for PFS compared with Endo alone, and vice versa, Endo alone has a better effect. (PFS: progression free survival; HR: hazard ratio; CrI: credible interval)

**Figure S2.** The SUCRA composite ranking rank of treatment regimens after Bayesian network meta-analysis of the random effects model for PFS[#1]

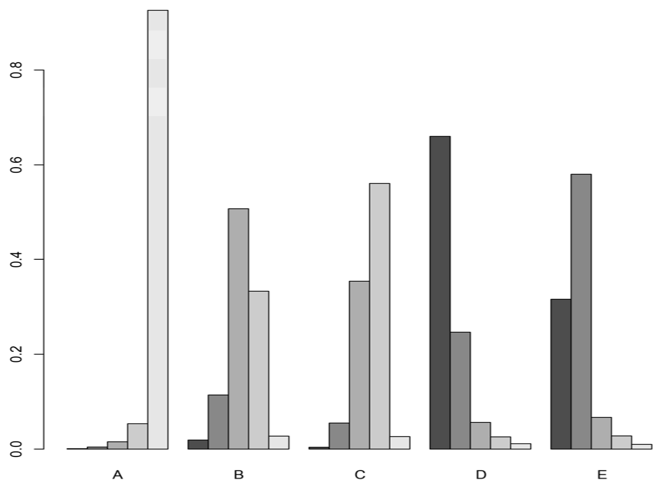


**Legend.** A: Endo; B: Her2-tki+Endo; C: Her2-mAb+Endo; D: Her2-mAb+Her2-mAb+Endo; E: Her2-mAb+Her2-tki+Endo. The horizontal axis indicates the ranking order of different treatment regimens, and the vertical axis indicates the cumulative probability of the corresponding ranking order. (A higher histogram represents a greater SUCRA, suggesting a greater likelihood of a higher ranking for that treatment option.)

**Figure S3.** The forest plot for the PFS[#2] after meta-analysis of the random-effects model Bayesian network

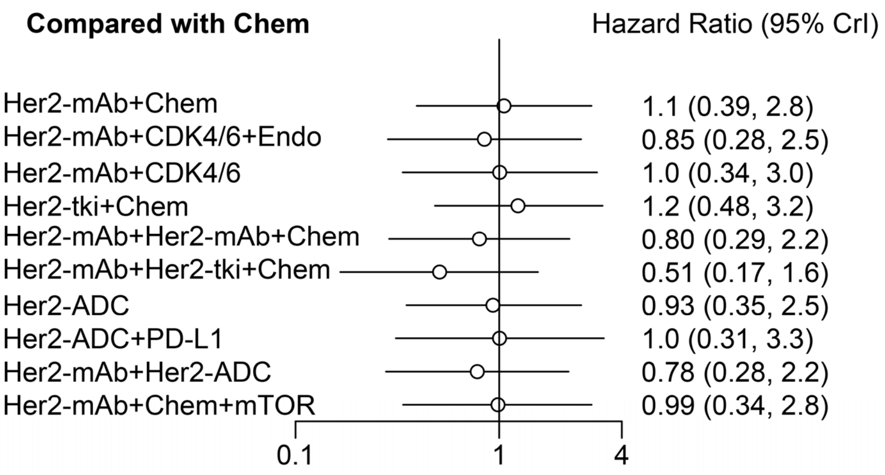


**Legend.** When HR and 95% CrI are less than 1, it indicates that the anti-HER2 combined with chemotherapy or CDK4/6 inhibitor has significant benefit for PFS compared with Chem alone, and vice versa, Chem alone has a better effect. (PFS: progression free survival; HR: hazard ratio; CrI: credible interval)

**Figure S4.** Random-effects model Bayesian network meta-analysis for PFS#2 after treatment regimen SUCRA integrated ranking scale

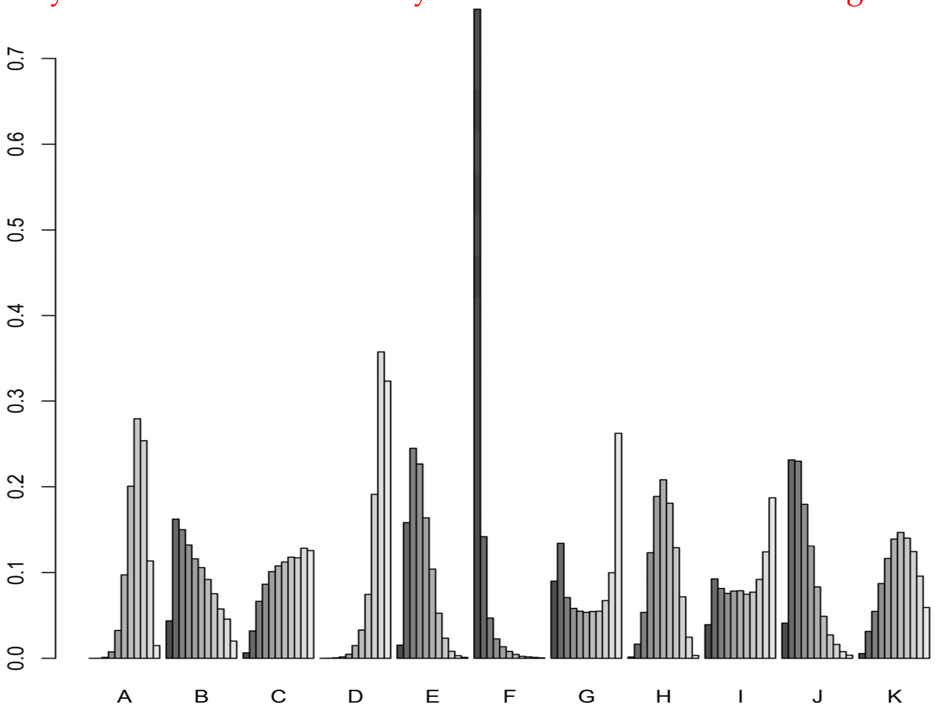


**Legend.** A: Her2-mAb+Chem; B: Her2-mAb+CDK4/6+Endo; C: Her2-mAb+CDK4/6; D: Her2-tki+Chem; E: Her2-mAb+ Her2-mAb+Chem; F: Her2-mAb+Her2-tki+Chem; G: Chem; H: Her2-ADC; I: Her2-ADC+PD-L1; J: Her2-mAb+Her2-ADC; K: Her2-mAb+Chem+mTOR. Note: The horizontal axis indicates the ranking of different treatment regimens The horizontal axis indicates the ranking of different treatment options, and the vertical axis indicates the cumulative probability of the corresponding ranking position. (A higher histogram indicates a higher SUCRA, suggesting a higher likelihood of a higher ranking of the treatment regimen.)

**Figure S5.** The forest plot for the OS[#1] after meta-analysis of the random-effects model Bayesian network

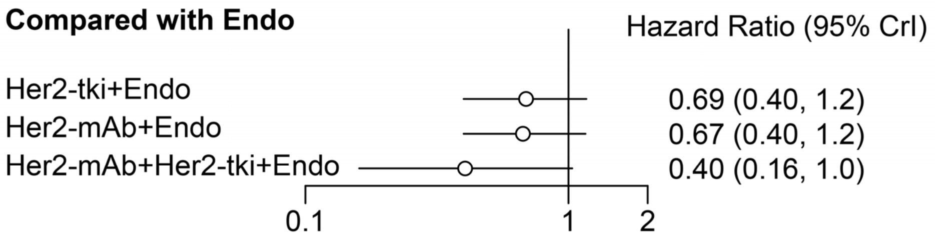


**Legend.** When HR and 95% CrI are less than 1, it indicates that the anti-HER2 combined with endocrine therapy has significant benefit for OS compared with Endo alone, and vice versa, Endo alone has a better effect。(OS:overall survival; HR: hazard ratio; CrI: credible interval)

**Figure S6.** Random-effects model Bayesian network meta-analysis for OS[#1] with the SUCRA composite ranking rank of treatment options

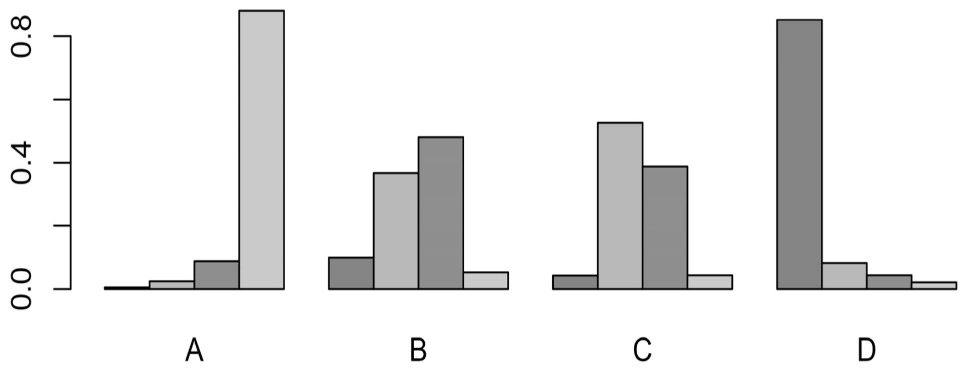


**Legend.** A: Endo; B: Her2-tki+Endo; C: Her2-mAb+Endo; D: Her2-mAb+Her2-tki+Endo. The horizontal axis indicates the ranking rank of different treatment options, and the vertical axis indicates the corresponding The vertical axis indicates the cumulative probability of the corresponding ranking. (A higher histogram indicates a greater SUCRA, suggesting a greater likelihood of a higher ranking for the treatment option.)

**Figure S7.** The forest plot for the OS[#2] after meta-analysis of the random-effects model Bayesian network

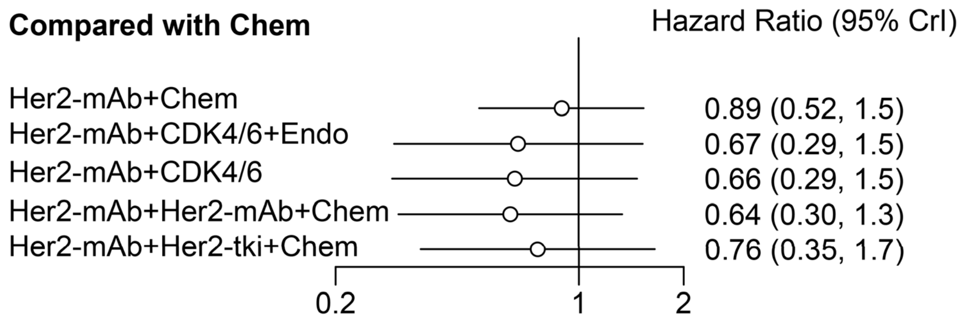


**Legend.** When HR and 95% CrI are less than 1, it indicates that the anti-HER2 combined with chemotherapy or CDK4/6 inhibitor has significant benefit for OS compared with Chem alone, and vice versa, Chem alone has a better effect. (OS: overall survival; HR: hazard ratio; CrI: credible interval)

**Figure S8.** Random-effects model Bayesian network meta-analysis for OS#2 after SUCRA combined ranking ranking of treatment regimens

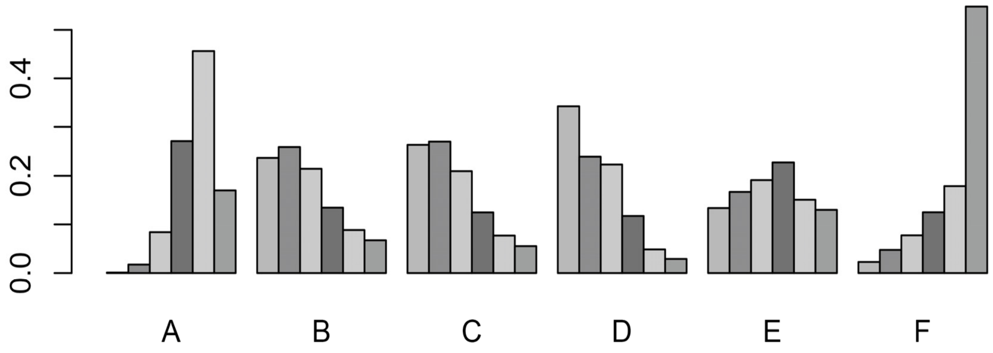


**Legend.** A: Her2-mAb+Chem; B: Her2-mAb+CDK4/6+Endo; C: Her2-mAb+CDK4/6; D: Her2-mAb+Her2-mAb+Chem; E: Her2 -mAb+Her2-mAb+Chem; F: Chem. The horizontal axis indicates the ranking order of different treatment regimens, and the vertical axis indicates the cumulative probability of the corresponding ranking order. (A higher histogram represents a greater SUCRA, suggesting a greater likelihood of a higher ranking for that treatment option.)

**Figure S9.** The forest plot for the ORR[#1] after meta-analysis of the random-effects model Bayesian network

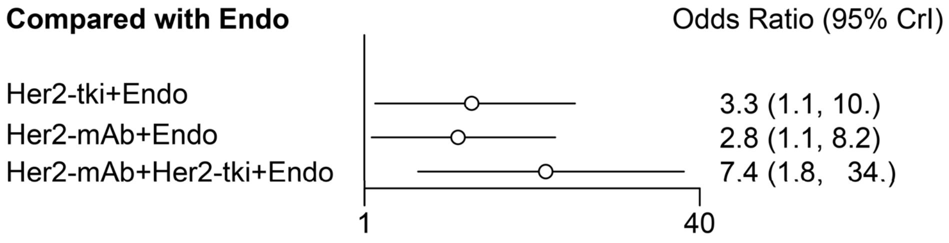


**Legend.** When OR and 95% CI are more than 1, it indicates that the anti-HER2 combined with endocrine therapy has significant benefit for ORR compared with Endo alone, and vice versa, Endo alone has a better effect. (ORR: objective response rate; OR: odds ratio; CI: confidence interval)

**Figure S10.** Random-effects model Bayesian network meta-analysis for ORR[#1] with the SUCRA composite ranking rank of treatment options

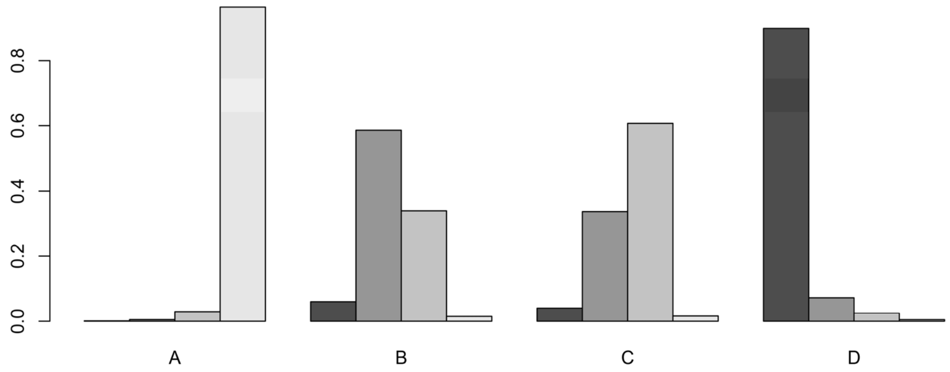


**Legend.** A: Endo; B: Her2-tki+Endo; C: Her2-mAb+Endo; D: Her2-mAb+Her2-tki+Endo. The horizontal axis indicates the ranking rank of different treatment options, and the vertical axis indicates the the cumulative probability of the corresponding ranking positions. (A higher histogram indicates a higher SUCRA, suggesting a higher probability of a higher ranking of the treatment option.)

**Figure S11.** The forest plot for the ORR[#2] after meta-analysis of the random-effects model Bayesian network

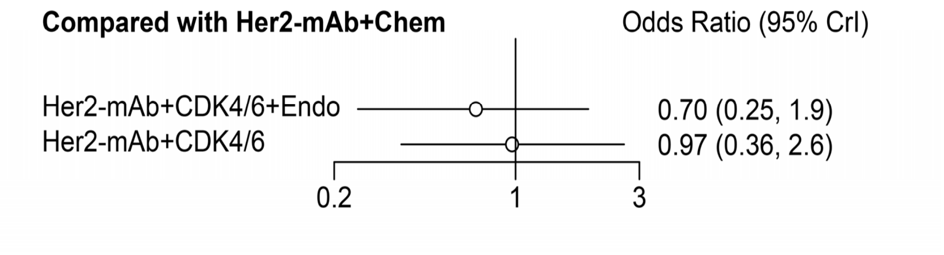


.

**Legend.** When OR and 95% CI are more than 1, it indicates that the anti-HER2 combined with chemotherapy or CDK4/6 inhibitor has significant benefit for ORR compared with Chem alone, and vice versa, Chem alone has a better effect. (ORR: objective response rate; OR: odds ratio; CI: confidence interval)

**Figure S12.** Random-effects model Bayesian network meta-analysis for ORR#2 with SUCRA composite ranking of treatment options

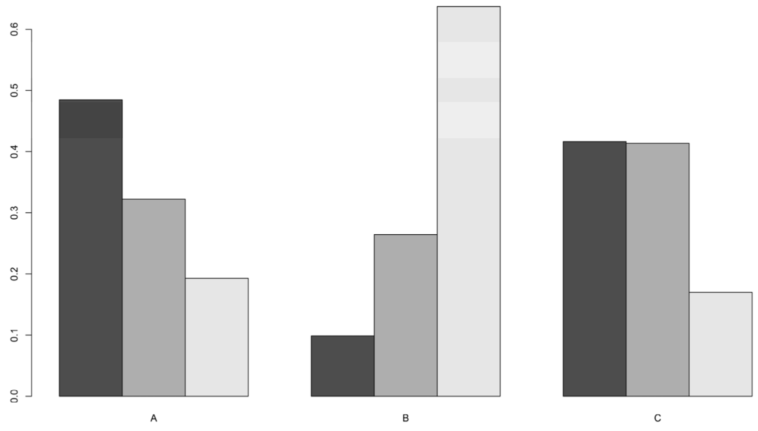


**Legend.** A: Her2-mAb+Chem; B: Her2-mAb+CDK4/6+Endo; C: Her2-mAb+CDK4/6. The horizontal axis indicates the ranking of different treatment options, and the vertical axis indicates the cumulative probability of the corresponding ranking. cumulative probability of the corresponding rank. (A higher histogram indicates a higher SUCRA, suggesting a higher likelihood of a higher ranking for the treatment option.)

**Figure S13.** The forest plot for the grade 3/4 adverse events after meta-analysis of the random-effects model Bayesian network

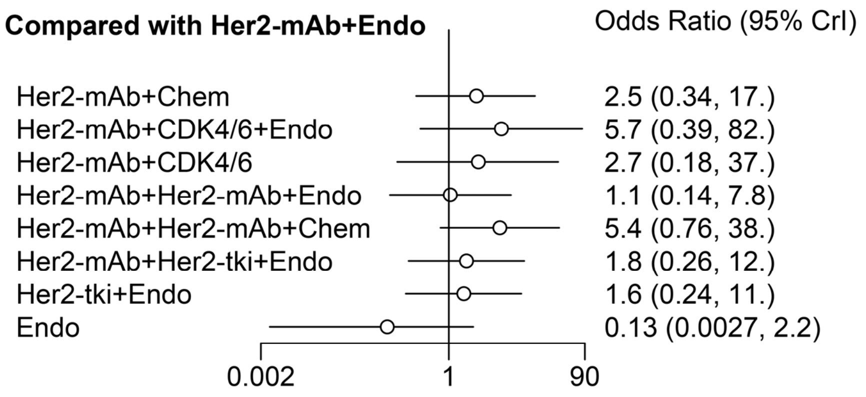


**Legend.** When OR and 95% CI are less than 1, it indicates that the anti-HER2 combination has significant benefit for reducing the incidence of grade 3/4 adverse events compared with Her2-mAb+Endo, and vice versa, Her2-mAb+Endo has a better effect.(OR: odds ratio; CI: confidence interval)

**Figure S14.** Random-effects model Bayesian network meta-analysis of treatment regimens after SUCRA composite ranking scale for grade 3/4 adverse events

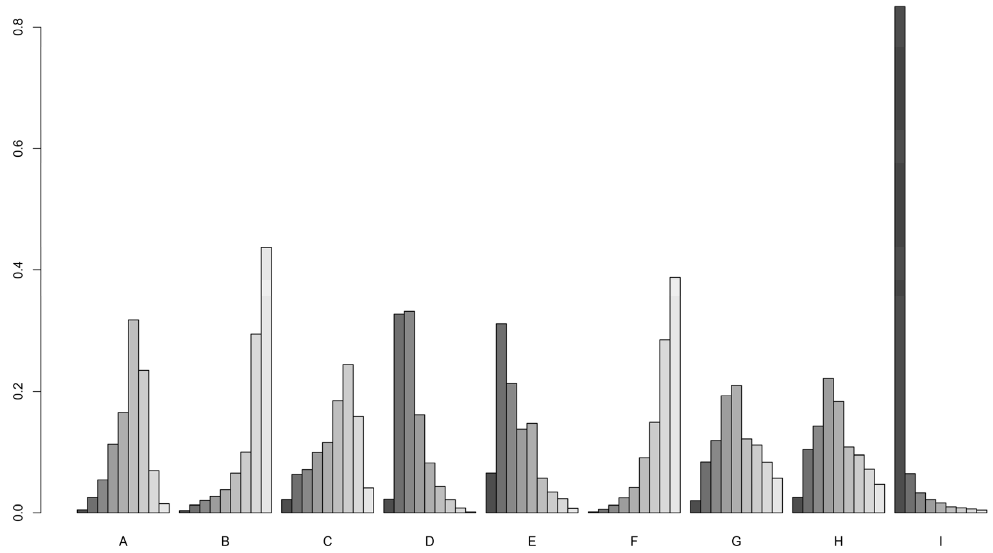


**Legend.** A: Her2-mAb+Chem; B: Her2-mAb+CDK4/6+Endo; C: Her2-mAb+CDK4/6; D: Her2-mAb+Endo; E: Her2- mAb+Her2-mAb+Endo; F: Her2-mAb+Her2-mAb+Chem; G: Her2-mAb+Her2-tki+Endo; H: Her2-tki+Endo; I: Endo. The horizontal axis indicates the ranking order of different treatment regimens, and the vertical axis indicates the cumulative probability of the corresponding ranking order. (A higher histogram represents a greater SUCRA, suggesting a greater likelihood of a higher ranking for that treatment option.)

## Supplementary Subgroup Analysis

**Table S3.** Bayesian network meta-analysis of PFS[#2] estimated HR between different treatment regimens (number of treatment lines equal to 1)

| | | | |
|---|---|---|---|
| **Her2-mAb+Chem** | 0.74 (0.54, 1.02) | 0.76 (0.49, 1.18) | 0.94 (0.61, 1.46) |
| 1.36 (0.98, 1.87) | **Her2-mAb+Her2-mAb+Chem** | 1.03 (0.6, 1.78) | 1.28 (0.74, 2.19) |
| 1.31 (0.84, 2.05) | 0.97 (0.56, 1.68) | **Her2-mAb+Her2-ADC** | 1.24 (0.79, 1.94) |
| 1.06 (0.68, 1.65) | 0.78 (0.46, 1.35) | 0.81 (0.51, 1.26) | **Her2-ADC** |

**Note.** HR<1 suggests that the column treatment regimen prolongs PFS compared to the row treatment regimen; HR>1 suggests that the column treatment regimen has poorer benefit for PFS than the row treatment regimen. Significant differences between the two groups are shown in bold.

**Table S4.** Bayesian network meta-analysis of PFS#2 estimated HR between different treatment regimens (number of treatment lines >1)

| | | | | | | | | | |
|---|---|---|---|---|---|---|---|---|---|
| **Her2-mAb+Chem** | 0.8 (0.39, 1.59) | 0.97 (0.49, 2) | 1.14 (0.76, 1.77) | 0.48 (0.23, 1.01) | 0.93 (0.3, 3) | 0.82 (0.38, 1.84) | 0.88 (0.29, 2.79) | 0.82 (0.41, 1.63) | 0.93 (0.48, 1.82) |
| 1.25 (0.63, 2.57) | **Her2-mAb+CDK4/6+Endo** | 1.21 (0.71, 2.24) | 1.43 (0.65, 3.35) | 0.6 (0.22, 1.7) | 1.17 (0.31, 4.55) | 1.02 (0.37, 3.06) | 1.1 (0.3, 4.34) | 1.02 (0.39, 2.82) | 1.17 (0.45, 3.14) |
| 1.04 (0.5, 2.02) | 0.83 (0.45, 1.42) | **Her2-mAb+CDK4/6** | 1.18 (0.51, 2.64) | 0.5 (0.17, 1.35) | 0.97 (0.25, 3.65) | 0.85 (0.29, 2.41) | 0.91 (0.24, 3.44) | 0.85 (0.31, 2.21) | 0.97 (0.36, 2.45) |
| 0.88 (0.56, 1.32) | 0.7 (0.3, 1.54) | 0.85 (0.38, 1.95) | **Her2-tki+Chem** | **0.42 (0.18, 0.98)** | 0.81 (0.28, 2.42) | 0.72 (0.37, 1.4) | 0.78 (0.27, 2.21) | 0.72 (0.32, 1.58) | 0.82 (0.37, 1.77) |
| 2.07 (0.99, 4.36) | 1.65 (0.59, 4.54) | 2.01 (0.74, 5.72) | **2.36 (1.02, 5.65)** | **Her2-mAb+Her2-tki+Chem** | 1.92 (0.5, 7.7) | 1.69 (0.59, 5.11) | 1.83 (0.49, 7.27) | 1.69 (0.62, 4.67) | 1.93 (0.72, 5.23) |
| 1.08 (0.33, 3.38) | 0.86 (0.22, 3.21) | 1.04 (0.27, 4.07) | 1.23 (0.41, 3.59) | 0.52 (0.13, 2) | **Chem** | 0.88 (0.25, 3.06) | 0.95 (0.21, 4.26) | 0.88 (0.23, 3.29) | 1 (0.26, 3.7) |
| 1.22 (0.54, 2.62) | 0.98 (0.33, 2.69) | 1.18 (0.42, 3.43) | 1.39 (0.71, 2.69) | 0.59 (0.2, 1.69) | 1.13 (0.33, 3.93) | **Her2-ADC** | 1.08 (0.47, 2.46) | 1 (0.34, 2.79) | 1.14 (0.4, 3.14) |
| 1.13 (0.36, 3.43) | 0.91 (0.23, 3.29) | 1.09 (0.29, 4.16) | 1.29 (0.45, 3.66) | 0.55 (0.14, 2.06) | 1.05 (0.23, 4.68) | 0.93 (0.41, 2.11) | **Her2-ADC+PD-L1** | 0.92 (0.24, 3.4) | 1.06 (0.28, 3.84) |
| 1.22 (0.61, 2.42) | 0.98 (0.35, 2.54) | 1.18 (0.45, 3.22) | 1.39 (0.63, 3.16) | 0.59 (0.21, 1.61) | 1.13 (0.3, 4.35) | 1 (0.36, 2.91) | 1.08 (0.29, 4.13) | **Her2-mAb+Her2-mAb+Chem** | 1.14 (0.44, 2.96) |
| 1.07 (0.55, 2.09) | 0.86 (0.32, 2.21) | 1.03 (0.41, 2.78) | 1.22 (0.57, 2.73) | 0.52 (0.19, 1.39) | 1 (0.27, 3.8) | 0.88 (0.32, 2.51) | 0.95 (0.26, 3.58) | 0.88 (0.34, 2.29) | **Her2-mAb+Chem+mTOR** |

**Note.** HR<1 suggests that the column treatment regimen prolongs PFS compared to the row treatment regimen; HR>1 suggests that the column treatment regimen has poorer benefit for PFS than the row treatment regimen. Significant differences between the two groups are shown in bold.

## Supplementary Sensitivity Analysis

**Table S5.** Fixed effects model Bayesian network meta-analysis of ORR[#1] between different treatment regimens Estimated OR

| | | | |
|---|---|---|---|
| **Endo** | **3.11 (1.71, 5.71)** | **2.62 (1.4, 5)** | **6.94 (3.29, 14.93)** |
| **0.32 (0.18, 0.59)** | **Her2-tki+Endo** | 0.84 (0.46, 1.54) | **2.23 (1.24, 4.08)** |
| **0.38 (0.2, 0.71)** | 1.19 (0.65, 2.15) | **Her2-mAb+Endo** | **2.65 (1.45, 4.93)** |
| **0.14 (0.07, 0.3)** | **0.45 (0.25, 0.81)** | **0.38 (0.2, 0.69)** | **Her2-mAb+Her2-tki+Endo** |

**Note.** The ORs and 95% CrI estimated for ORR[#1] between treatment regimens are shown in the table above for each column treatment group compared to each row treatment group. OR>1 indicates that the column treatment regimen improves the ORR of patients compared to the row treatment regimen; OR<1 indicates that the column treatment regimen has a poorer benefit on ORR compared to the row treatment regimen. Two groups with significant differences are shown in bold for comparison.

**Table S6.** Fixed effects model Bayesian network meta-analysis of ORR[#2] between different treatment regimens to estimate OR

| | | |
|---|---|---|
| **Her2-mAb+Chem** | 0.7 (0.3, 1.62) | 0.96 (0.43, 2.15) |
| 1.43 (0.62, 3.34) | **Her2-mAb+CDK4/6+Endo** | 1.36 (0.65, 2.94) |
| 1.05 (0.47, 2.3) | 0.73 (0.34, 1.55) | **Her2-mAb+CDK4/6** |

**Note.** The ORs and 95% CrI estimated for ORR[#2] between treatment regimens are shown in the table above for each column treatment group compared to each row treatment group. OR>1 indicates that the column treatment regimen improves the ORR of patients compared to the row treatment regimen; OR<1 indicates that the column treatment regimen has a poorer benefit on ORR compared to the row treatment regimen. Two groups with significant differences are shown in bold for comparison.

**Table S7.** Fixed-effects model Bayesian network meta-analysis of PFS#1 estimated HRs between different treatment regimens

| | | | | |
|---|---|---|---|---|
| **Endo** | **0.65 (0.51, 0.82)** | **0.69 (0.55, 0.86)** | **0.38 (0.22, 0.64)** | **0.45 (0.32, 0.64)** |
| **1.55 (1.21, 1.97)** | **Her2-tki+Endo** | 1.06 (0.83, 1.36) | **0.58 (0.34, 1)** | **0.7 (0.51, 0.95)** |
| **1.46 (1.16, 1.83)** | 0.94 (0.74, 1.21) | **Her2-mAb+Endo** | **0.55 (0.34, 0.89)** | **0.66 (0.48, 0.9)** |
| **2.65 (1.56, 4.49)** | **1.72 (1, 2.93)** | **1.82 (1.13, 2.92)** | **Her2-mAb+Her2-mAb+Endo** | 1.19 (0.68, 2.11) |
| **2.22 (1.57, 3.15)** | **1.44 (1.06, 1.96)** | **1.52 (1.11, 2.09)** | 0.84 (0.47, 1.48) | **Her2-mAb+Her2-tki+Endo** |

**Note.** The estimated HR and 95% CrI for PFS#1 between treatment regimens are shown in the table above, for each column treatment group compared to each row treatment group. HR<1 indicates that the column treatment regimen prolongs PFS compared to the row treatment regimen; HR>1 indicates that the column treatment regimen has poorer benefit for PFS compared to the row treatment regimen. Significant differences between the two groups are shown in bold.

**Table S8.** Fixed-effects model Bayesian network meta-analysis of PFS#2 estimated ORs between different treatment regimens

| | | | | | | | | | | |
|---|---|---|---|---|---|---|---|---|---|---|
| **Her2-mAb+Chem** | 0.81 (0.53, 1.25) | 0.95 (0.62, 1.46) | 1.17 (0.97, 1.42) | **0.76 (0.64, 0.89)** | **0.48 (0.31, 0.75)** | 0.96 (0.37, 2.49) | 0.88 (0.71, 1.09) | 0.95 (0.51, 1.77) | **0.74 (0.56, 0.97)** | 0.93 (0.72, 1.2) |
| 1.23 (0.8, 1.89) | **Her2-mAb+CDK4/6+Endo** | 1.18 (0.81, 1.72) | 1.44 (0.9, 2.31) | 0.93 (0.59, 1.48) | 0.59 (0.32, 1.1) | 1.18 (0.42, 3.35) | 1.08 (0.67, 1.75) | 1.17 (0.55, 2.49) | 0.91 (0.54, 1.51) | 1.15 (0.7, 1.89) |
| 1.05 (0.68, 1.61) | 0.85 (0.58, 1.24) | **Her2-mAb+CDK4/6** | 1.23 (0.77, 1.97) | 0.79 (0.5, 1.26) | **0.5 (0.27, 0.93)** | 1 (0.35, 2.85) | 0.92 (0.57, 1.49) | 1 (0.47, 2.13) | 0.77 (0.46, 1.28) | 0.98 (0.59, 1.61) |
| 0.85 (0.7, 1.03) | 0.69 (0.43, 1.11) | 0.81 (0.51, 1.3) | **Her2-tki+Chem** | **0.65 (0.5, 0.83)** | **0.41 (0.25, 0.66)** | 0.82 (0.32, 2.08) | **0.75 (0.62, 0.91)** | 0.81 (0.44, 1.5) | **0.63 (0.46, 0.85)** | 0.79 (0.58, 1.09) |
| **1.32 (1.12, 1.57)** | 1.07 (0.68, 1.7) | 1.26 (0.8, 2) | **1.55 (1.2, 2)** | **Her2-mAb+Her2-mAb+Chem** | 0.63 (0.4, 1.02) | 1.27 (0.48, 3.33) | 1.16 (0.89, 1.53) | 1.26 (0.66, 2.39) | 0.97 (0.7, 1.35) | 1.23 (0.9, 1.67) |
| **2.08 (1.34, 3.23)** | 1.69 (0.91, 3.12) | **1.99 (1.07, 3.67)** | **2.44 (1.51, 3.95)** | 1.58 (0.98, 2.52) | **Her2-mAb+Her2-tki+Chem** | 1.99 (0.7, 5.69) | **1.83 (1.12, 2.99)** | 1.98 (0.92, 4.24) | 1.53 (0.91, 2.58) | **1.94 (1.16, 3.22)** |
| 1.05 (0.4, 2.71) | 0.85 (0.3, 2.4) | 1 (0.35, 2.82) | 1.23 (0.48, 3.11) | 0.79 (0.3, 2.08) | 0.5 (0.18, 1.44) | **Chem** | 0.92 (0.35, 2.38) | 0.99 (0.32, 3.04) | 0.77 (0.29, 2.05) | 0.97 (0.36, 2.6) |
| 1.14 (0.92, 1.41) | 0.92 (0.57, 1.49) | 1.08 (0.67, 1.75) | **1.33 (1.1, 1.61)** | 0.86 (0.66, 1.13) | **0.55 (0.33, 0.89)** | 1.09 (0.42, 2.82) | **Her2-ADC** | 1.08 (0.6, 1.94) | 0.84 (0.63, 1.11) | 1.06 (0.76, 1.47) |
| 1.05 (0.56, 1.97) | 0.85 (0.4, 1.82) | 1 (0.47, 2.14) | 1.23 (0.67, 2.29) | 0.8 (0.42, 1.52) | 0.51 (0.24, 1.09) | 1.01 (0.33, 3.09) | 0.93 (0.52, 1.67) | **Her2-ADC+PD-L1** | 0.78 (0.41, 1.48) | 0.98 (0.5, 1.93) |
| **1.36 (1.03, 1.79)** | 1.1 (0.66, 1.84) | 1.3 (0.78, 2.16) | **1.59 (1.18, 2.16)** | 1.03 (0.74, 1.42) | 0.65 (0.39, 1.1) | 1.3 (0.49, 3.46) | 1.19 (0.9, 1.58) | 1.29 (0.67, 2.46) | **Her2-mAb+Her2-ADC** | 1.26 (0.87, 1.84) |
| 1.08 (0.83, 1.39) | 0.87 (0.53, 1.44) | 1.02 (0.62, 1.69) | 1.26 (0.92, 1.74) | 0.81 (0.6, 1.11) | **0.52 (0.31, 0.86)** | 1.03 (0.38, 2.76) | 0.95 (0.68, 1.32) | 1.02 (0.52, 2) | 0.79 (0.54, 1.15) | **Her2-mAb+Chem+mTOR** |

**Note.** The estimated HR and 95% CrI for PFS#2 between treatment regimens are shown in the table above, for each column treatment group compared to each row treatment group. HR<1 indicates that the column treatment regimen prolongs PFS compared to the row treatment regimen; HR>1 indicates that the column treatment regimen has poorer benefit for PFS compared to the row treatment regimen. Significant differences between the two groups are shown in bold.

**Table S9.** Fixed effects model Bayesian network meta-analysis of OS#1 estimated ORs between different treatment regimens

| | | | |
|---|---|---|---|
| **Endo** | **0.7 (0.53, 0.92)** | **0.67 (0.5, 0.88)** | **0.4 (0.22, 0.74)** |
| **1.44 (1.09, 1.89)** | **Her2-tki+Endo** | 0.95 (0.68, 1.33) | 0.57 (0.3, 1.09) |
| **1.5 (1.14, 1.98)** | 1.05 (0.75, 1.46) | **Her2-mAb+Endo** | 0.6 (0.35, 1.04) |
| **2.5 (1.35, 4.61)** | 1.74 (0.92, 3.3) | 1.66 (0.96, 2.88) | **Her2-mAb+Her2-tki+Endo** |

**Note.** The estimated HR and 95% CrI for OS#1 between treatment regimens are shown in the table above, for each column treatment group compared to each row treatment group. HR<1 indicates that the column treatment regimen prolongs patient OS compared to the row treatment regimen; HR>1 indicates that the column treatment regimen has a poorer benefit on OS compared to the row treatment regimen. Significant differences between the two groups are shown in bold.

**Table S10.** Fixed-effects model Bayesian network meta-analysis of OS#2 estimated ORs between different treatment regimens

| | | | | | |
|---|---|---|---|---|---|
| **Her2-mAb+Chem** | 0.75 (0.47, 1.2) | 0.73 (0.47, 1.15) | **0.71 (0.53, 0.95)** | 0.85 (0.59, 1.24) | 1.12 (0.77, 1.62) |
| 1.33 (0.83, 2.14) | **Her2-mAb+CDK4/6+Endo** | 0.98 (0.63, 1.54) | 0.95 (0.55, 1.65) | 1.14 (0.62, 2.07) | 1.49 (0.82, 2.71) |
| 1.36 (0.87, 2.14) | 1.02 (0.65, 1.6) | **Her2-mAb+CDK4/6** | 0.97 (0.57, 1.66) | 1.16 (0.65, 2.08) | 1.52 (0.85, 2.73) |
| **1.4 (1.05, 1.88)** | 1.05 (0.6, 1.83) | 1.03 (0.6, 1.77) | **Her2-mAb+Her2-mAb+Chem** | 1.2 (0.74, 1.92) | 1.57 (0.97, 2.52) |
| 1.17 (0.81, 1.7) | 0.88 (0.48, 1.6) | 0.86 (0.48, 1.55) | 0.84 (0.52, 1.34) | **Her2-mAb+Her2-tki+Chem** | 1.31 (0.77, 2.22) |
| 0.9 (0.62, 1.3) | 0.67 (0.37, 1.22) | 0.66 (0.37, 1.18) | 0.64 (0.4, 1.03) | 0.76 (0.45, 1.29) | **Chem** |

**Note.** The estimated HR and 95% CrI for OS#2 between treatment regimens are shown in the table above, for each column treatment group compared to each row treatment group. HR<1 indicates that the column treatment regimen prolongs patient OS compared to the row treatment regimen; HR>1 indicates that the column treatment regimen has a poorer benefit on OS compared to the row treatment regimen. Significant differences between the two groups are shown in bold.

**Table S11.** Fixed-effects model Bayesian network meta-analysis of the incidence of grade 3/4 adverse events between different treatment regimens to estimate OR

| | | | | | | | | |
|---|---|---|---|---|---|---|---|---|
| **Her2-mAb+Chem** | **2.28 (1.18, 4.42)** | 1.09 (0.57, 2.06) | **0.4 (0.18, 0.85)** | **0.42 (0.19, 0.9)** | **2.15 (1.1, 4.29)** | 0.72 (0.27, 1.87) | 0.66 (0.25, 1.72) | **0.05 (0, 0.54)** |
| **0.44 (0.23, 0.85)** | **Her2-mAb+CDK4/6+Endo** | **0.48 (0.25, 0.88)** | **0.18 (0.06, 0.47)** | **0.19 (0.07, 0.5)** | 0.95 (0.37, 2.42) | **0.31 (0.1, 1)** | **0.29 (0.09, 0.92)** | **0.02 (0, 0.26)** |
| 0.92 (0.49, 1.75) | **2.1 (1.13, 3.94)** | **Her2-mAb+CDK4/6** | **0.37 (0.14, 0.99)** | 0.39 (0.14, 1.06) | 1.99 (0.79, 5.04) | 0.66 (0.21, 2.1) | 0.6 (0.19, 1.93) | **0.05 (0, 0.54)** |
| **2.49 (1.18, 5.42)** | **5.67 (2.11, 15.69)** | **2.69 (1.01, 7.32)** | **Her2-mAb+Endo** | 1.05 (0.45, 2.45) | **5.35 (2.54, 11.82)** | **1.78 (1, 3.25)** | 1.63 (0.91, 2.98) | 0.14 (0, 1.19) |
| **2.36 (1.11, 5.18)** | **5.4 (1.99, 15.02)** | 2.57 (0.95, 6.98) | 0.95 (0.41, 2.22) | **Her2-mAb+Her2-mAb+Endo** | **5.08 (2.4, 11.27)** | 1.69 (0.61, 4.78) | 1.55 (0.56, 4.37) | 0.13 (0, 1.32) |
| **0.46 (0.23, 0.91)** | 1.06 (0.41, 2.69) | 0.5 (0.2, 1.27) | **0.19 (0.08, 0.39)** | **0.2 (0.09, 0.42)** | **Her2-mAb+Her2-mAb+Chem** | **0.33 (0.12, 0.87)** | **0.3 (0.11, 0.8)** | **0.02 (0, 0.25)** |
| 1.4 (0.53, 3.7) | **3.19 (1, 10.29)** | 1.52 (0.48, 4.82) | 0.56 (0.31, 1) | 0.59 (0.21, 1.65) | **3 (1.15, 8.02)** | **Her2-mAb+Her2-tki+Endo** | 0.92 (0.53, 1.59) | **0.08 (0, 0.72)** |
| 1.52 (0.58, 4.04) | **3.48 (1.09, 11.2)** | 1.65 (0.52, 5.28) | 0.61 (0.34, 1.1) | 0.64 (0.23, 1.8) | **3.29 (1.26, 8.73)** | 1.09 (0.63, 1.9) | **Her2-tki+Endo** | **0.08 (0, 0.79)** |
| **18.63 (1.85, 616.64)** | **42.73 (3.85, 1492.13)** | **20.26 (1.84, 700.07)** | 7.34 (0.84, 231.61) | 7.85 (0.76, 268.05) | **40 (3.98, 1358.26)** | **13.22 (1.4, 430.37)** | **12.16 (1.27, 387.06)** | **Endo** |

**Note.** The ORs and 95% CrIs estimated for the incidence of grade 3/4 adverse events between treatment regimens are shown in the table above for each column treatment group compared with each row treatment group. OR<1 indicates that the column treatment regimen decreased the incidence of grade ≥3 adverse events in patients compared with the row treatment regimen; OR>1 indicates that the column treatment regimen increased the incidence of grade ≥3 adverse events in patients compared with the row treatment regimen. Significant differences are shown in bold for comparison of the two groups.

## Supplementary Small-study Effects

**Figure S15.** Comparative-corrected funnel plots for ORR[#1]

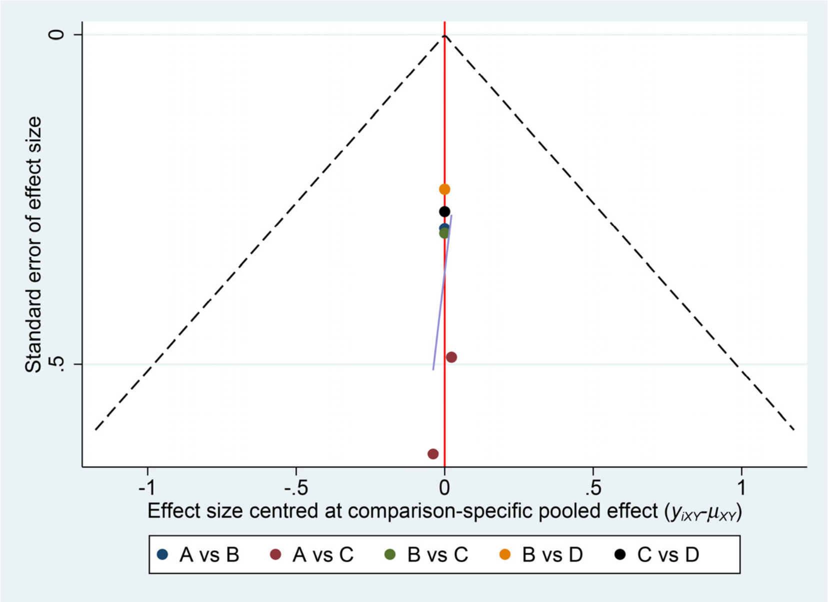


**Legend.** A: Endo; B: Her2-tki+Endo; C: Her2-mAb+Endo; D: Her2-mAb+Her2-tki+Endo. The central vertical red line indicates that the effect sizes specific to this study are not different from the corresponding comparison-specific effect estimates, and the line through the vertical red line is the regression line. Different direct comparisons are indicated by different colored scatter points. (ORR: objective response rate)

**Figure S16.** Comparative-corrected funnel plots for ORR[#2]

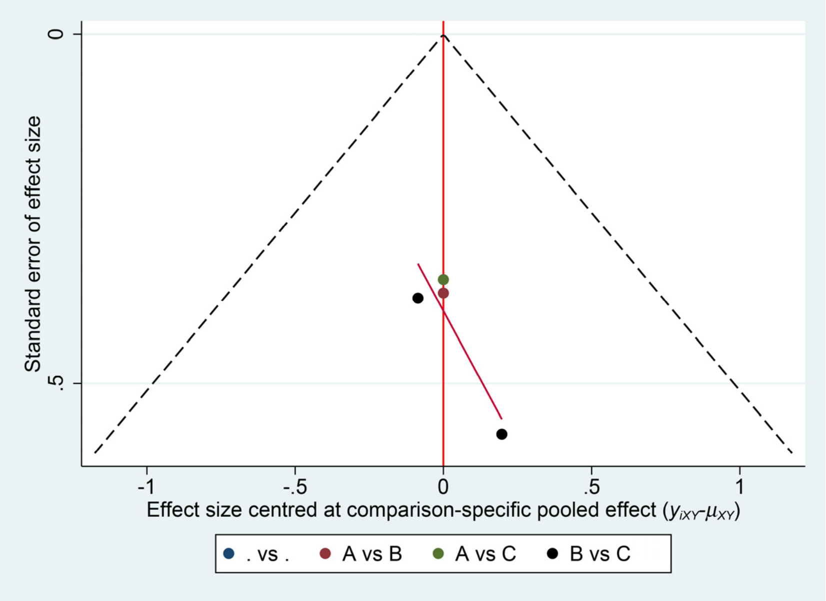


**Legend.** A: Her2-mAb+Chem; B: Her2-mAb+CDK4/6+Endo; C: Her2-mAb+CDK4/6. The central vertical red line indicates that the effect sizes specific to this study are not different from the corresponding comparison-specific effect estimates, and the line through the vertical red line is the regression line. Different direct comparisons are indicated by different colored scatter points. (ORR: objective response rate)

**Figure S17.** Comparative-corrected funnel plots for PFS[#1]

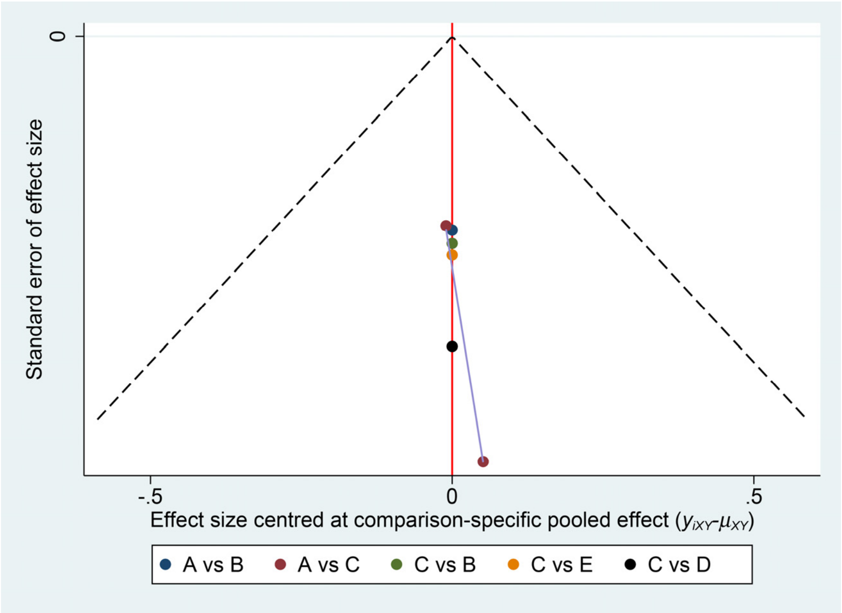


**Legend.** A: Endo; B: Her2-tki+Endo; C: Her2-mAb+Endo; D: Her2-mAb+Her2-mAb+Endo; E: Her2-mAb+Her2-tki+Endo. The central vertical red line indicates that the effect sizes specific to this study are not different from the corresponding comparison-specific effect estimates, and the line through the vertical red line is the regression line. Different direct comparisons are indicated by different colored scatter. (PFS: progression free survival)

**Figure S18.** Comparative-corrected funnel plots for PFS[#2]

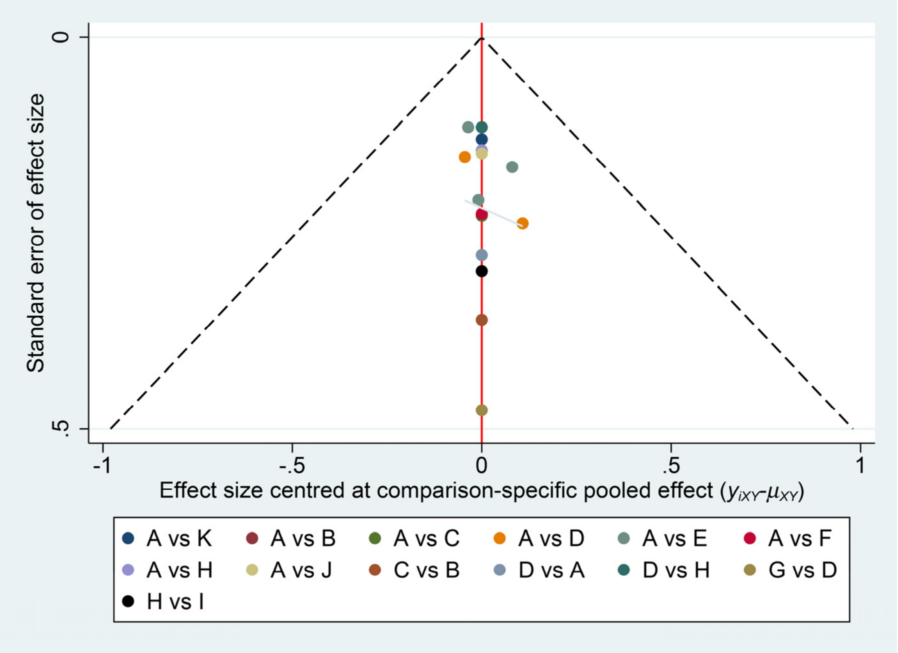


**Legend.** A: Her2-mAb+Chem; B: Her2-mAb+CDK4/6+Endo; C: Her2-mAb+CDK4/6; D: Her2-tki+Chem; E: Her2-mAb+Her2-mAb+Chem; F: Her2-mAb+Her2-tki+Chem; G: Chem; H: Her2-ADC; I: Her2-ADC+PD-L1; J: Her2-mAb+Her2-ADC; K: Her2-mAb+Chem+mTOR. The central vertical red line indicates that the effect sizes specific to this study are not different from the corresponding comparison-specific effect estimates, and the line through the vertical red line is the regression line. Different direct comparisons are indicated by different colored scatter. (PFS: progression free survival)

**Figure S19.** Comparative-corrected funnel plots for OS[#1]

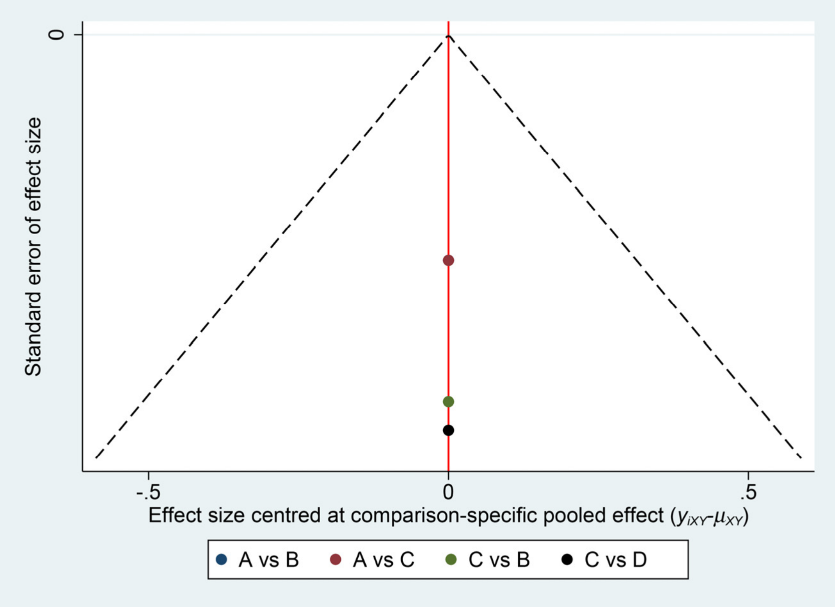


**Legend.** A: Endo; B: Her2-tki+Endo; C: Her2-mAb+Endo; D: Her2-mAb+Her2-tki+Endo. The central vertical red line indicates that the effect sizes specific to this study are not different from the corresponding comparison-specific effect estimates, and the line through the vertical red line is the regression line. Different direct comparisons are indicated by different colored scatter. (OS: overall survival)

**Figure S20.** Comparative-corrected funnel plots for OS[#2]

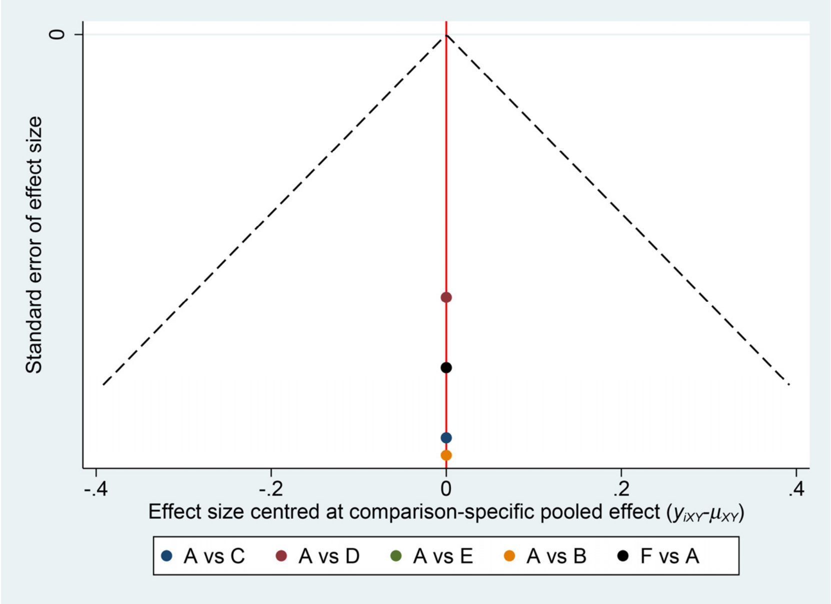


**Legend.** A: Her2-mAb+Chem; B: Her2-mAb+CDK4/6+Endo; C: Her2-mAb+CDK4/6; D: Her2-mAb+Her2-mAb+Chem; E: Her2-mAb+Her2-tki+Chem; F: Chem. The central vertical red line indicates that the effect sizes specific to this study are not different from the corresponding comparison-specific effect estimates, and the line through the vertical red line is the regression line. Different direct comparisons are indicated by different colored scatter. (OS: overall survival)

**Figure S21.** Comparative-corrected funnel plots for grade 3/4 adverse events

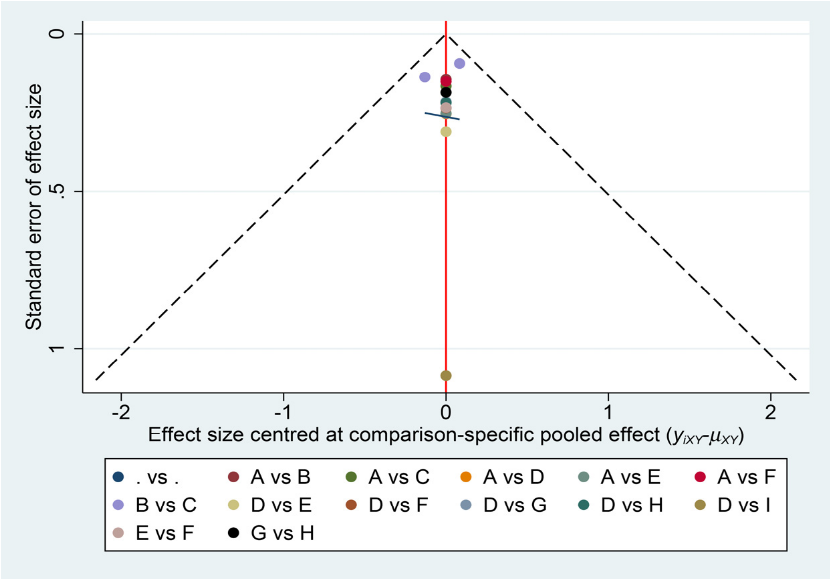


**Legend.** A: Her2-mAb+Chem; B: Her2-mAb+CDK4/6+Endo; C: Her2-mAb+CDK4/6; D: Her2-mAb+Endo; E: Her2-mAb+Her2-mAb+Endo; F: Her2-mAb+Her2-mAb+Chem; G: Her2-mAb+Her2-tki+Endo; H: Her2-tki+Endo; I: Endo. The central vertical red line indicates that the effect sizes specific to this study are not different from the corresponding comparison-specific effect estimates, and the line through the vertical red line is the regression line. Different direct comparisons are indicated by different colored scatter.
